# Synthesis and structural characterization of a hydrated sodium–caesium tetra­cosa­tungstate(VI), Na_5_Cs_19_[W_24_O_84_]·21H_2_O

**DOI:** 10.1107/S2056989024004778

**Published:** 2024-05-31

**Authors:** Gauthier Deblonde, Ian Colliard

**Affiliations:** a Physical and Life Sciences Directorate, Glenn T. Seaborg Institute, Lawrence Livermore National Laboratory, Livermore, California 94550, USA; Vienna University of Technology, Austria

**Keywords:** crystal structure, polyoxidometalates, isopolytungstates, toroidal structures, counter-ion conversion.

## Abstract

Crystal formation of penta­sodium nona­deca­cesium tetra­cosa­tungstate(VI) heneikosahydrate, Na_5_Cs_19_[W_24_O_84_]·21H_2_O, was successfully achieved by the conversion of [H_2_W_12_O_42_]^10−^ through the addition of excess Cs^+^. The crystal structure comprising the toroidal isopolyoxidometalate is presented, as well as its Raman spectrum. Na_5_Cs_19_(H_2_O)_21_W_24_O_84_ crystallizes in the rhombohedral space group *R*  with an obverse centering. The title compound represents the addition of a new member to the isopolytungstate family with mixed alkali counter-ions and contains rarely observed five-coordinate tungsten(VI) atoms in the [W_24_O_84_]^24−^ anion (site symmetry *C*_3*i*_) arising from the conversion mediated by Cs^+^ counter-ions.

## Abstract 2

The isopolytungstate(VI) [W_24_O_84_]^24–^ anion in the hydrated mixed-alkali isopolytungstate exhibits mol­ecular *C*_3*i*_ symmetry.

## 1. Chemical context

Isopolytungstates are a class of polyoxidometalates (POMs). They are sometimes described as anionic mol­ecular oxides of *d*-block metals with no additional elements, aside from the counter-ions. These discrete metal-oxygen mol­ecules are often composed of octa­hedrally coordinated metal ions, bridged by oxygen ions. The elements forming POMs are typically high-oxidation state metals, such as W^VI^, Mo^VI^, Nb^V^, Ta^V^, *etc.* (Pope, 1983 ▸). Polytungstate formation is often seen as a result of controlling hydrolysis and condensation reactions, which otherwise results in an oxide. Structural and compositional variability of polytungstates increases with the incorporation of other elements, such as phosphates or silicates (Wang *et al.*, 2024 ▸), which are named heteropolytungstates. Nevertheless, factors such as counter-ions and concentration can and have influenced the formation of metal-oxido species, becoming a more popular avenue to obtain new compounds with new structures (Antonio *et al.*, 2009 ▸). The number of isopolytungstate general structures has so far been limited to a handful, *e.g*. [W_4_O_16_]^8–^, [W_6_O_19_]^2–^, [W_7_O_24_]^6–^, [W_10_O_32_]^4–^, [H_2_W_12_O_40_]^6–^, [H_2_W_12_O_42_]^10–^ and others (Liu *et al.*, 2018 ▸). The corresponding compounds have generated growing inter­est since their original discovery because of their diverse chemical and physical properties, making them of considerable value in various fields including catalysis, optics, quantum devices, materials sciences, medicine, and beyond.

## 2. Structural commentary

**Figure 1:**
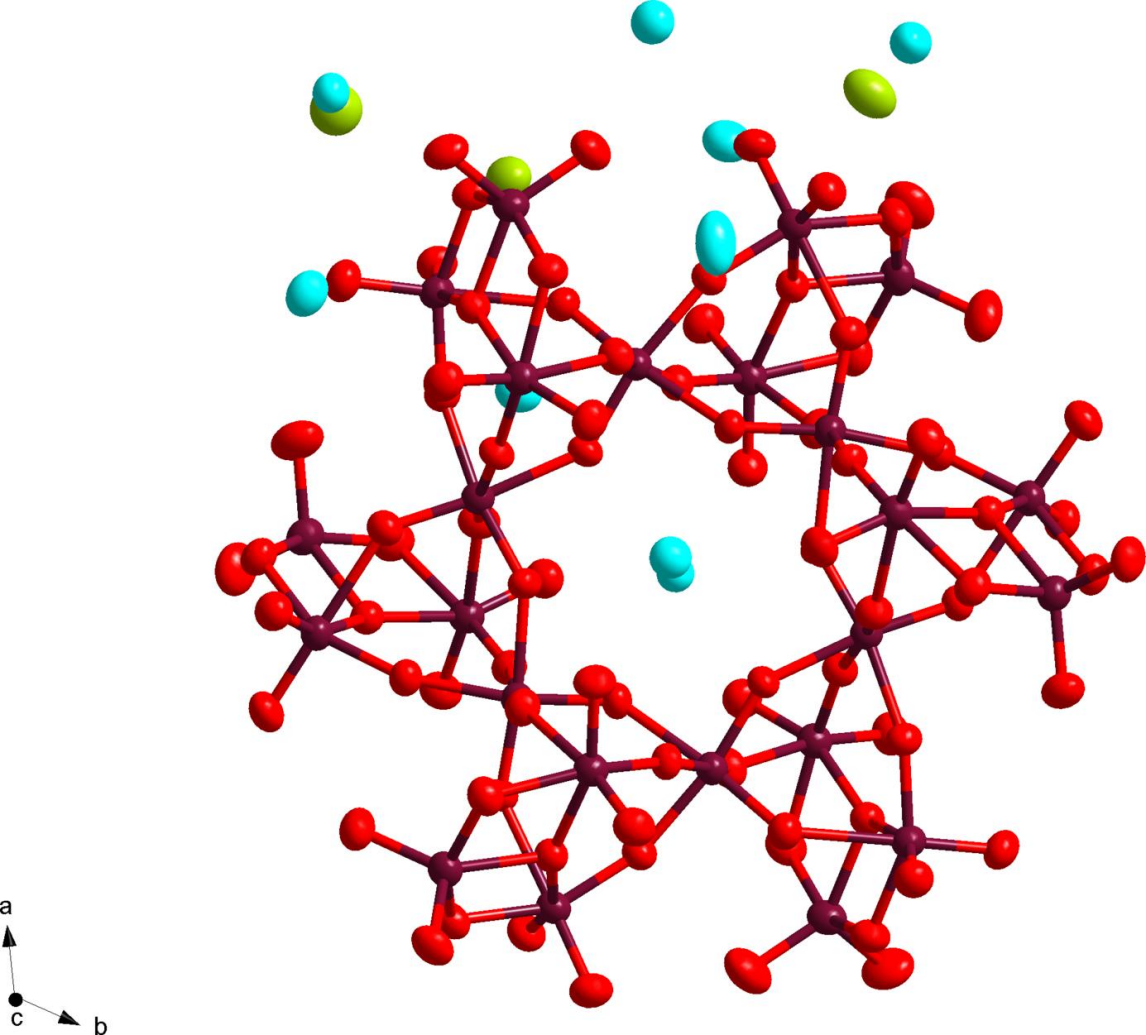
Ellipsoid and stick representation of the [W_24_O_84_]^24–^ polyoxidotungstate anion at the 50% probability level. The shown counter-ions and O atoms of solvent water mol­ecules represent those from the asymmetric unit (see Fig. 2 ▸). Color code: W in maroon, Cs in light blue, Na in green, and O in red.

**Figure 2:**
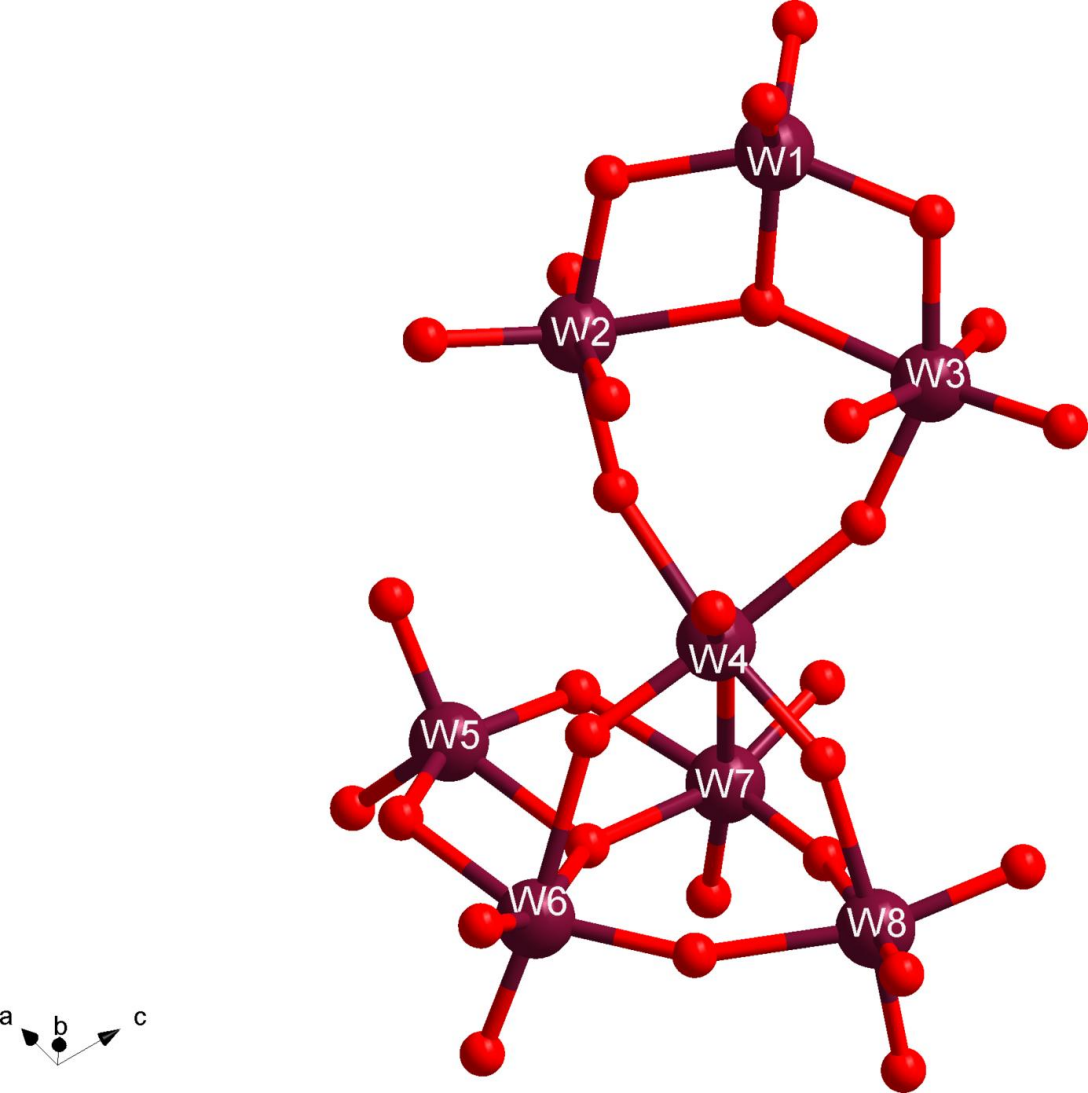
Ball-and-stick representation of the asymmetric unit, comprised of two [W_4_O_14_]^4–^ building blocks, showing the five-coordinate W1 and W5 atoms. The tip of the tetra­mer (W4) connects to the next tetra­mer, which W8 then repeats; the whole asymmetric unit needs to be repeated three times to complete the entire anion. Counter-ions, O atoms of solvent water mol­ecules, and oxygen labels are omitted for clarity. Color code: W in maroon and O in red.

The hydrated mixed alkali isopolytungstate, fully formulated as Na_5_Cs_19_(H_2_O)_21_W_24_O_84_, crystallizes in the trigonal space group *R* 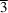 with an obverse rhombohedral centering. This compound is based on the tetra­cosa­tungstate(VI) core: [W_24_O_84_]^24–^ (Fig. 1 ▸). Before detailing the inter­connectivity, description of the symmetry elements present will aid in the construction of the core anion. The [W_24_O_84_]^24–^ isopolytungstate anion can best be described by the point group *S*_6_ otherwise known as *C*_3*i*_. The anion thus only has threefold improper rotation symmetry around an inversion point that is outside the anion, within the central void (Fig. 1 ▸). Considering only the asymmetric unit of the anion, an octa­meric [W_8_O_28_]^8–^ subunit is present, disregarding the counter-ions at the moment. Applying the threefold improper rotation on the asymmetric unit creates the complete anion. An alternate view can be seen through the deconstruction of the asymmetric unit into smaller substructures, [W_4_O_14_]^4–^, *i.e.* two tetra­mers fused together. The smaller tungstate subunit can be seen as three six-coordinate W atoms (W2, W3, and W4, or W6, W7, and W8), with a W—O bond length range of 1.771 (10)–2.293 (9) Å (average 1.956 Å), plus one five-coordinate W atom (W1 or W5) with a bond length range of 1.732 (12)–2.008 (9) Å (average 1.867 Å). W1 and W5 uniquely feature two terminal O atoms with bond lengths between 1.732 (12) and 1.765 (9) Å. In contrast, the six-coordinate W atoms have only one terminal O atom, with the remaining five oxygen atoms bridging to neighboring W atoms. The W atoms are arranged in a kite shape with the three six-coordinate W atoms (W2, W3, and W4) making the tip and the five-coordinate W atom (W1) making the cap (Fig. 2 ▸). The tetra­meric subunits are linked up through the ‘tip-most’ tungstate, *i.e.* W4 or W8. Each of these W atoms bridges to the neighboring tetra­mer; W4 is bound to W6, W7, and W8. The other ‘tip-most’ W atom, W8 thus does the same (bridged to W2, W3, and W4). Thus, the anion takes on a hexa­gonal shape with the five-coordinate W atoms on the periphery, alternating above or below a plane created by the central W4 and W8 atoms. Two unique cesium counter-ions (Cs1 and Cs2) fill the void left by the anion both with a twelve coordination and a Cs—O bond length range of 3.139 (9)–3.657 (9) Å. These Cs atoms are actually located on the same axis as the threefold improper rotation symmetry operation (1/3, 2/3, *z*, Wyckoff letter *c*) representing a special symmetry position for the space group, consistent with the obverse centering. The remaining Cs^+^ counter-ions fill in the space made by linking the tetra­meric units in a pinwheel structure and connect to the neighboring [W_24_O_84_]^24–^ anion making the long-range structure. Cs6 is the only counter-ion located at the edge of the unit cell on another threefold rotation axis (1, 1, *z*). The sodium counter-ions, on the other hand, feature higher hydration (in terms of bound water O atoms) than connectivity to the O atoms of the tungstate anions, in line with the typical higher polytungstate inter­actions with larger alkali cations, relative to small ones (Misra *et al.*, 2020 ▸). Four of the sodium counter-ions can be located in between [W_24_O_84_]^24–^ units, making a sodium-based tetra­meric [Na_4_(H_2_O)_15_] unit with an Na—O bond length range of 2.380 (16)–2.508 (19) Å (more in the next section). The sodium counter-ion Na2 and Cs8 are also at special symmetry positions (2/3, 1/3, *z*). The remaining Na^+^ counter-ion, Na3, is disordered over three positions and links water mol­ecules along the *c* axis.

## 3. Supra­molecular features

**Figure 3:**
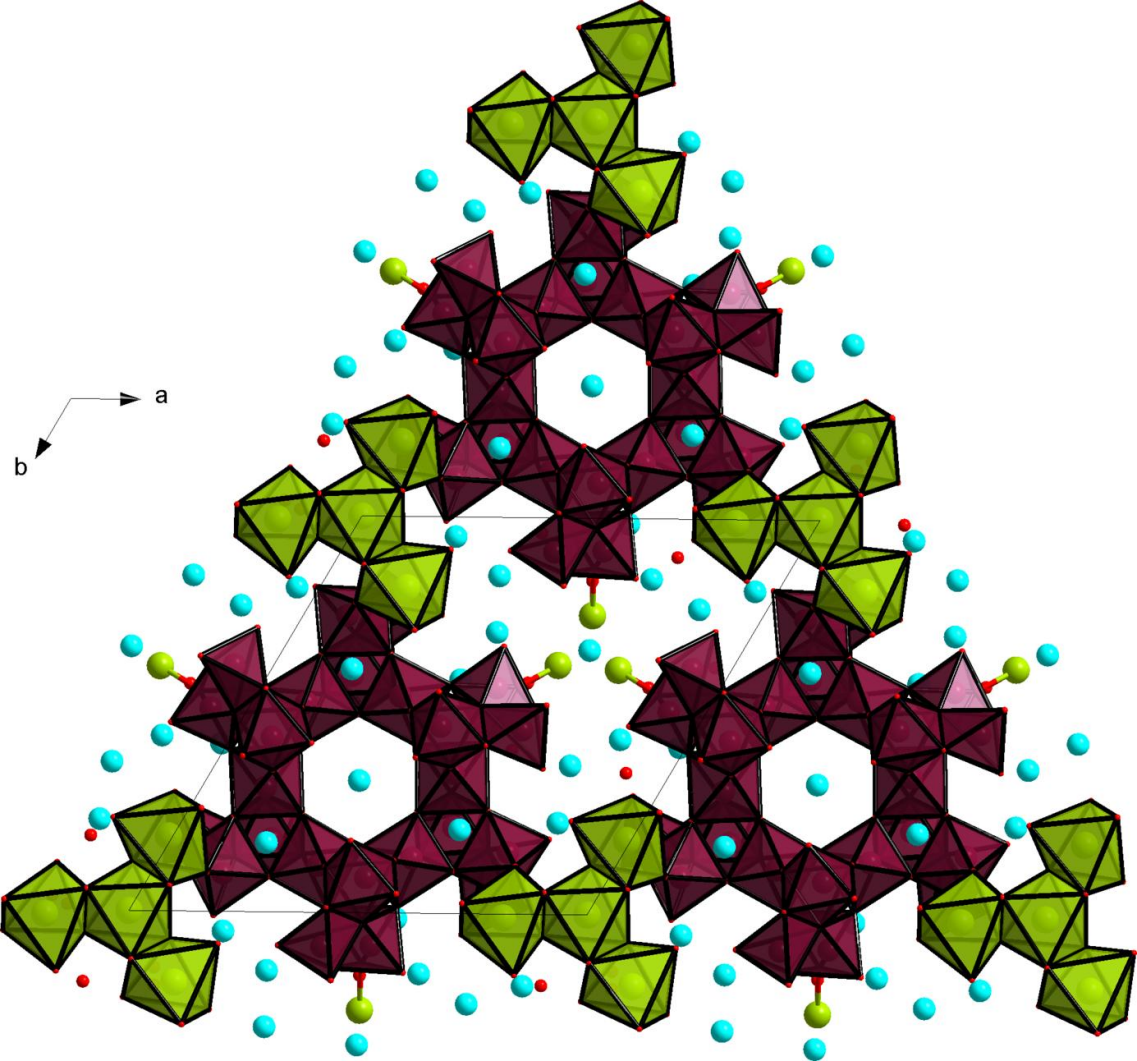
Projection of the crystal structure along [001] in a polyhedral representation of the [W_24_O_84_]^24–^ (maroon) and [Na_5_(H_2_O)_18_]^5+^ (green) units, showcasing the long-range structure formation, connectivity and unit cell. Cs^+^ counter-ions are in blue, Na^+^ in green.

The unique structure formation for [W_24_O_84_]^24–^ is mainly attributed to the Na^+^ counter-ions. The section above described the formation of the [Na_4_(H_2_O)_15_] unit, where the Na^+^ cations are triangularly arranged, with one central Na^+^ cation linked to three other Na^+^ cations (Fig. 3 ▸). As such, each corner of the triangle formed by the Na^+^ centers links to one [W_24_O_84_]^24–^ anion, hence arranging the [W_24_O_84_]^24–^ anions into the hexa­gonal sheets. The Cs^+^ counter-ions additionally link [W_24_O_84_]^24–^ along the *c* axis (Fig. 3 ▸).

Since water H atoms could not be located, details of possible hydrogen-bonding inter­actions were approximated by calculating the shortest O⋯O distances for the water mol­ecules, which were measured to be on average 2.8 Å (Table 1 ▸).

## 4. Database survey

A search of the Inorganic Crystal Structure Database (ICSD-408188, release 2024.1 version 5.2.0; Zagorac *et al.*, 2019 ▸) and the Cambridge Structural Database (CSD, accessed on April 2024; Groom *et al.*, 2016 ▸) for closely related sodium cesium polyoxidotungstates was performed. The first search involved a unit cell search (*a* = *b* = 17.998 Å, *c* = 62.879, *α* = *β* = 90°, and *γ* = 120°, with tolerance of 10% each). With a rhombohedral centering no results were found, with a primitive setting six results were found, none of which contained any tungstates. Therefore, a second search was conducted based on the general anion formula ‘W_24_O_84_’ with the option to allow other elements in the mol­ecule. Two matches were received: first is Cs_24_[W_24_O_84_]·26H_2_O, which features the anion with all Cs^+^ counter-ions, but with lower point group symmetry (*C*_i_) in comparison with the title compound (deposition number 408188, reference code 1725526; Brüdgam *et al.*, 1998 ▸). Therein the authors suggest that the anion could only be isolated with Cs^+^ counter-ions, with no other alkali. However, the new structure confirms the essence of Cs^+^ to template the [W_24_O_84_]^24–^ anion, as well as the role Na^+^ can have in long-range structure formation. The second, (C_12_H_35_As_2_Mn_3_N_2_O_105_P_4_W_24_^9–^)_*n*_·6(C_2_H_8_N^+^)·7(H_2_O)·1.2(K^+^)·2.4(Li^+^)·0.6(Cl^−^), deposition number 2251558, reference code CODJUE, can best be described as a fragment of the [P_5_W_30_O_110_]^14–^ anion (Iftikhar *et al.*, 2024 ▸). Further extensive searches with varying compositions and unit-cell tolerances were performed, but no additional structures were found.

## 5. Synthesis and crystallization

All materials herein were obtained and used as received, with no need for further purification: NaCl (>=99.9%), NaCH_3_COO (>=99.9%), cesium chloride (>99.99%), and Na_2_WO_4_·2H_2_O (>=99%). All solutions were prepared using deionized water purified by reverse osmosis cartridge system (>= 18.2 MΩ.cm). All experiments were performed in a temperature-controlled room (295 K). Na_2_WO_4_·2H_2_O was dissolved in 50 ml of boiling water. The pH of the solution was adjusted to 7.2 with 6 *M* HCl added dropwise. After boiling the solution for 1 h the volume reduced to 20 ml. Then, 5 g NaCl were added, and the solution was left to cool to 278 K. Sodium paratungstate A crystallized out and was collected. The resulting sodium paratungstate A, formulated as Na_10_H_2_W_12_O_42_·*n*H_2_O (10 g), was dissolved in 20 ml of boiling water. After complete dissolution, 10 g of CsCl were added, and the solution was left to cool. Immediately upon reaching room temperature, crystals of the title compound appeared.

**Figure 4:**
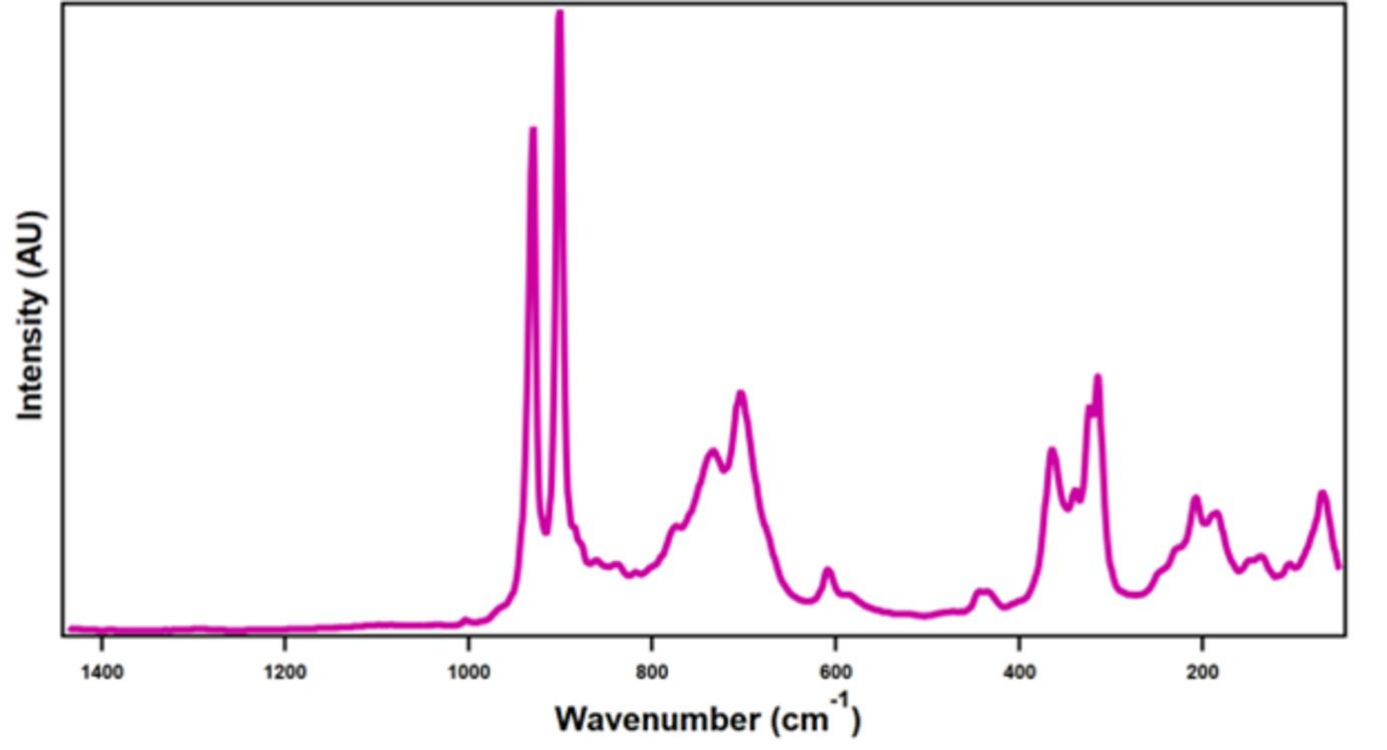
Solid-state Raman spectrum of Na_5_Cs_19_[W_24_O_84_]·21H_2_O.

Raman spectra were collected using a Senterra II confocal Raman microscope (Bruker), equipped with high resolution gratings (1200 lines mm^−1^) and a 532 nm laser source (operated at 15 mW), and a TE-cooled CCD detector. The shown spectrum is based on the average of at least 2–5 different spots per sample, each spot analysis consisting of 2 binned 16 scans. The integration time was set to 2000 ms per scan. No damage to the sample was observed due to the laser irradiation. Selected Raman data (cm^−1^): *ν*(W=O^t^) 930 and 901, and *ν*(O—W—O) 703, 364, 314 and 207 (Fig. 4 ▸).

## 6. Refinement

Crystal data, data collection and structure refinement details are summarized in Table 2 ▸. All atoms were refined anisotropically, except for two O atoms of water mol­ecules (O1*W* and O4*W*). In addition, Na3 was first refined with a free site occupation factor which converged to a value of 0.333 to which it was fixed for charge balance in the final refinement cycles. Hydrogen atoms of the water mol­ecules could not be located and are not part of the model but are considered in the formula and other crystallographic data. Due to the high atomic number (*Z*) for W and Cs atoms, high residual electron-difference peaks of about 10% of *Z Å* ^−3^ remained, which is considered as inconspicuous (Massa & Gould, 2004 ▸); the highest residual peak is located at 0.452 Å from the nearest W atom.

## Supplementary Material

Crystal structure: contains datablock(s) I. DOI: 10.1107/S2056989024004778/wm5717sup1.cif

Structure factors: contains datablock(s) I. DOI: 10.1107/S2056989024004778/wm5717Isup2.hkl

CCDC reference: 2345067

Additional supporting information:  crystallographic information; 3D view; checkCIF report

## Figures and Tables

**Table 1:** Water O atoms: nearest neighbor atoms (Å) for possible hydrogen-bonding inter­actions

O1*W*⋯O28	2.72 (3)
O1*W*⋯O28^i^	2.97 (3)
O2*W*⋯O21^i^	2.927 (17)
O2*W*⋯O27^i^	2.867 (19)
O3*W*⋯O10	2.690 (15)
O3*W*⋯O25^ii^	2.739 (15)
O4*W*⋯O23	2.775 (17)
O5*W*⋯O6*W*	3.074 (19)
O5*W*⋯O10^ii^	2.765 (16)
O5*W*⋯O12^iii^	2.745 (18)
O6*W*⋯O8	2.935 (15)
O6*W*⋯O8^iv^	2.991 (15)
O6*W*⋯O11	2.989 (16)
O7*W*⋯O8	3.259 (18)

Symmetry codes: (i) −*y* + 1, *x* − *y* + 1, *z*; (ii) *x* − *y* + 1, *x*, −*z* + 1; (iii) −*y* + 2, *x* − *y* + 1, *z*; (iv) −*x* + *y* + 1, −*x* + 1, *z*.

**Table 2:** Experimental details

Crystal data	
Chemical formula	Na_5_Cs_19_[W_24_O_84_]·21H_2_O
*M*_r_	8774.97
Crystal system, space group	Trigonal, *R* 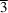
Temperature (K)	298
*a*, *c* (Å)	18.0071 (3), 62.9142 (9)
*V* (Å^3^)	17667.1 (6)
*Z*	6
Radiation type	Mo *K*α
μ (mm^−1^)	29.24
Crystal size (mm)	0.02 × 0.02 × 0.01

Data collection	
Diffractometer	ROD, Synergy Custom DW system, Pilatus 300K
Absorption correction	Multi-scan (*CrysAlis PRO*; Rigaku OD, 2019 ▸)
*T*_min_, *T*_max_	0.001, 0.007
No. of measured, independent and observed [*I* > 2σ(*I*)] reflections	30484, 8019, 7262
*R*_int_	0.033
(sin θ/λ)_max_ (Å^−1^)	0.625

Refinement	
*R*[*F* ^2^ > 2σ(*F* ^2^)], *wR*(*F* ^2^), *S*	0.062, 0.170, 1.08
No. of reflections	8019
No. of parameters	456
H-atom treatment	H-atom parameters not defined
Δρ_max_, Δρ_min_ (e Å^−3^)	5.28, −1.53

Computer programs: *CrysAlis PRO* (Rigaku OD, 2019 ▸), *SHELXT* (Sheldrick, 2015*a* ▸), *SHELXL* (Sheldrick, 2015*b* ▸) and *OLEX2* (Dolomanov *et al.*, 2009 ▸).

## supplementary crystallographic information

### Crystal data

**Table:**

Na_5_Cs_19_[W_24_O_84_]·21H_2_O	*D*_x_ = 4.949 Mg m^−^^3^
*M**_r_* = 8774.97	Mo *K*α radiation, λ = 0.71073 Å
Trigonal, *R*3	Cell parameters from 12610 reflections
*a* = 18.0071 (3) Å	θ = 3.5–31.6°
*c* = 62.9142 (9) Å	µ = 29.24 mm^−^^1^
*V* = 17667.1 (6) Å^3^	*T* = 298 K
*Z* = 6	Rhombohedral, clear colourless
*F*(000) = 22548	0.02 × 0.02 × 0.01 mm

### Data collection

**Table:**

ROD, Synergy Custom DW system, Pilatus 300K diffractometer	7262 reflections with *I* > 2σ(*I*)
Detector resolution: 5.8140 pixels mm^-1^	*R*_int_ = 0.033
ω scans	θ_max_ = 26.4°, θ_min_ = 3.5°
Absorption correction: multi-scan (CrysAlisPro; Rigaku OD, 2019)	*h* = −22→22
*T*_min_ = 0.001, *T*_max_ = 0.007	*k* = −20→22
30484 measured reflections	*l* = −78→78
8019 independent reflections	

### Refinement

**Table:**

Refinement on *F*^2^	0 restraints
Least-squares matrix: full	H-atom parameters not defined
*R*[*F*^2^ > 2σ(*F*^2^)] = 0.062	*w* = 1/[σ^2^(*F*_o_^2^) + (0.1323*P*)^2^ + 146.9834*P*] where *P* = (*F*_o_^2^ + 2*F*_c_^2^)/3
*wR*(*F*^2^) = 0.170	(Δ/σ)_max_ = 0.002
*S* = 1.08	Δρ_max_ = 5.28 e Å^−^^3^
8019 reflections	Δρ_min_ = −1.53 e Å^−^^3^
456 parameters	

### Special details

**Table:**

Geometry. All esds (except the esd in the dihedral angle between two l.s. planes) are estimated using the full covariance matrix. The cell esds are taken into account individually in the estimation of esds in distances, angles and torsion angles; correlations between esds in cell parameters are only used when they are defined by crystal symmetry. An approximate (isotropic) treatment of cell esds is used for estimating esds involving l.s. planes.

### Fractional atomic coordinates and isotropic or equivalent isotropic displacement parameters (Å^2^)

**Table:**

	*x*	*y*	*z*	*U*_iso_*/*U*_eq_	Occ. (<1)
W1	0.75322 (4)	0.95534 (3)	0.45841 (2)	0.04877 (16)	
W2	0.74914 (3)	0.84611 (3)	0.41728 (2)	0.04257 (16)	
W3	0.57837 (3)	0.77373 (3)	0.46489 (2)	0.04252 (16)	
W4	0.52779 (3)	0.64798 (3)	0.41276 (2)	0.03935 (15)	
W5	0.62832 (3)	0.52673 (3)	0.37414 (2)	0.04459 (16)	
W6	0.44888 (3)	0.51802 (3)	0.36310 (2)	0.04151 (15)	
W7	0.51118 (3)	0.42927 (3)	0.41456 (2)	0.04238 (16)	
W8	0.31337 (3)	0.45144 (3)	0.41362 (2)	0.03987 (16)	
Cs1	0.333333	0.666667	0.44521 (2)	0.0516 (3)	
Cs2	0.333333	0.666667	0.38025 (3)	0.0594 (4)	
Cs3	0.45537 (6)	0.50778 (7)	0.46622 (2)	0.0616 (3)	
Cs4	0.68039 (11)	0.75734 (8)	0.35676 (2)	0.0910 (4)	
Cs5	0.81936 (7)	0.77587 (8)	0.47655 (2)	0.0713 (3)	
Cs6	1.000000	1.000000	0.43671 (5)	0.0908 (7)	
Cs7	0.89457 (7)	0.70886 (7)	0.40700 (2)	0.0677 (3)	
Cs8	0.666667	0.333333	0.39181 (3)	0.0574 (4)	
Cs9	0.43603 (8)	0.28384 (7)	0.35495 (2)	0.0637 (3)	
Na1	0.6763 (4)	0.5222 (4)	0.46411 (11)	0.0680 (15)	
Na2	0.666667	0.333333	0.4601 (2)	0.090 (4)	
Na3	0.9123 (14)	0.9508 (16)	0.3695 (3)	0.081 (6)	0.3333
O1	0.5059 (5)	0.4707 (5)	0.38389 (13)	0.0416 (17)	
O1W	0.5091 (16)	0.7593 (16)	0.3353 (4)	0.140 (7)*	
O2	0.4861 (6)	0.7600 (7)	0.47844 (16)	0.053 (2)	
O2W	0.7058 (10)	0.9593 (12)	0.3648 (3)	0.101 (5)	
O3	0.3627 (5)	0.4801 (5)	0.38316 (13)	0.0412 (17)	
O3W	0.6409 (7)	0.5930 (7)	0.49145 (18)	0.062 (2)	
O4	0.8169 (6)	0.8096 (7)	0.42717 (17)	0.056 (2)	
O4W	0.7190 (9)	0.6333 (9)	0.4392 (2)	0.082 (3)*	
O5	0.6915 (6)	0.8350 (5)	0.44706 (14)	0.0428 (18)	
O5W	0.8200 (9)	0.5687 (10)	0.4768 (2)	0.092 (4)	
O6	0.8373 (8)	0.9769 (9)	0.47624 (18)	0.072 (3)	
O6W	0.7211 (8)	0.4579 (9)	0.4383 (2)	0.072 (3)	
O7	0.2437 (5)	0.4952 (5)	0.40723 (14)	0.0416 (17)	
O7W	0.6122 (10)	0.3994 (9)	0.4864 (2)	0.087 (4)	
O8	0.5467 (6)	0.4313 (7)	0.44085 (16)	0.052 (2)	
O9	0.6524 (6)	0.7424 (6)	0.41085 (14)	0.0450 (18)	
O10	0.6179 (7)	0.7267 (7)	0.48315 (15)	0.054 (2)	
O11	0.6289 (6)	0.4826 (7)	0.40195 (14)	0.0473 (19)	
O12	0.7505 (10)	1.0493 (8)	0.4548 (2)	0.077 (3)	
O13	0.4066 (6)	0.4229 (6)	0.41866 (14)	0.0456 (18)	
O14	0.5724 (6)	0.5622 (6)	0.35439 (14)	0.0473 (19)	
O15	0.5347 (6)	0.6913 (5)	0.44449 (13)	0.0426 (17)	
O16	0.7241 (7)	0.6243 (7)	0.37647 (19)	0.061 (3)	
O17	0.5209 (6)	0.6283 (6)	0.38466 (14)	0.0464 (19)	
O18	0.7944 (7)	0.8887 (7)	0.39216 (17)	0.056 (2)	
O19	0.4140 (5)	0.5798 (5)	0.41757 (14)	0.0436 (18)	
O20	0.6509 (7)	0.4598 (7)	0.35819 (15)	0.055 (2)	
O21	0.2371 (6)	0.3418 (6)	0.40834 (15)	0.0480 (19)	
O22	0.4777 (7)	0.3239 (7)	0.40671 (18)	0.058 (2)	
O23	0.5588 (6)	0.5716 (6)	0.42080 (14)	0.0445 (18)	
O24	0.2958 (6)	0.4485 (6)	0.44194 (14)	0.0482 (19)	
O25	0.6541 (7)	0.8955 (6)	0.47625 (14)	0.052 (2)	
O26	0.8071 (6)	0.9605 (6)	0.43191 (15)	0.048 (2)	
O27	0.4054 (7)	0.4304 (7)	0.34583 (16)	0.055 (2)	
O28	0.4333 (7)	0.5931 (6)	0.34822 (15)	0.051 (2)	

### Atomic displacement parameters (Å^2^)

**Table:**

	*U*^11^	*U*^22^	*U*^33^	*U*^12^	*U*^13^	*U*^23^
W1	0.0510 (3)	0.0422 (3)	0.0481 (3)	0.0196 (2)	−0.0043 (2)	−0.00508 (19)
W2	0.0368 (3)	0.0406 (3)	0.0477 (3)	0.0174 (2)	0.00026 (18)	−0.00177 (18)
W3	0.0447 (3)	0.0424 (3)	0.0412 (3)	0.0223 (2)	0.00020 (18)	−0.00170 (17)
W4	0.0371 (3)	0.0370 (3)	0.0432 (3)	0.01792 (19)	−0.00016 (17)	−0.00228 (17)
W5	0.0416 (3)	0.0472 (3)	0.0467 (3)	0.0235 (2)	0.00246 (18)	−0.00100 (19)
W6	0.0425 (3)	0.0425 (3)	0.0410 (3)	0.0224 (2)	−0.00064 (17)	0.00015 (17)
W7	0.0414 (3)	0.0437 (3)	0.0459 (3)	0.0242 (2)	−0.00031 (18)	0.00199 (18)
W8	0.0362 (3)	0.0391 (3)	0.0439 (3)	0.01857 (19)	0.00058 (17)	0.00079 (17)
Cs1	0.0495 (5)	0.0495 (5)	0.0557 (8)	0.0248 (2)	0.000	0.000
Cs2	0.0610 (6)	0.0610 (6)	0.0561 (8)	0.0305 (3)	0.000	0.000
Cs3	0.0548 (5)	0.0752 (6)	0.0535 (5)	0.0316 (4)	−0.0020 (3)	0.0003 (4)
Cs4	0.1294 (12)	0.0574 (6)	0.0695 (7)	0.0342 (7)	0.0080 (7)	0.0053 (5)
Cs5	0.0609 (6)	0.0827 (7)	0.0675 (6)	0.0338 (5)	0.0038 (4)	0.0031 (5)
Cs6	0.0607 (7)	0.0607 (7)	0.151 (2)	0.0303 (3)	0.000	0.000
Cs7	0.0682 (6)	0.0660 (6)	0.0697 (6)	0.0342 (5)	−0.0057 (4)	−0.0104 (4)
Cs8	0.0519 (5)	0.0519 (5)	0.0685 (9)	0.0259 (2)	0.000	0.000
Cs9	0.0808 (7)	0.0650 (6)	0.0562 (5)	0.0446 (5)	−0.0051 (4)	−0.0002 (4)
Na1	0.066 (4)	0.072 (4)	0.069 (4)	0.037 (3)	0.000 (3)	−0.009 (3)
Na2	0.095 (6)	0.095 (6)	0.079 (8)	0.048 (3)	0.000	0.000
Na3	0.067 (12)	0.090 (15)	0.061 (11)	0.022 (11)	−0.004 (9)	0.002 (10)
O1	0.040 (4)	0.042 (4)	0.044 (4)	0.021 (4)	0.002 (3)	0.001 (3)
O2	0.053 (5)	0.055 (5)	0.052 (5)	0.027 (4)	0.009 (4)	0.004 (4)
O2W	0.078 (9)	0.128 (13)	0.095 (10)	0.051 (9)	0.012 (8)	0.035 (9)
O3	0.034 (4)	0.042 (4)	0.041 (4)	0.015 (3)	0.005 (3)	0.004 (3)
O3W	0.060 (6)	0.064 (6)	0.063 (6)	0.031 (5)	−0.003 (5)	−0.005 (5)
O4	0.041 (5)	0.062 (6)	0.071 (6)	0.028 (5)	−0.006 (4)	−0.008 (5)
O5	0.041 (4)	0.039 (4)	0.047 (4)	0.019 (4)	0.001 (3)	0.000 (3)
O5W	0.069 (8)	0.103 (10)	0.062 (7)	0.012 (7)	0.001 (6)	−0.006 (7)
O6	0.062 (7)	0.079 (8)	0.056 (6)	0.021 (6)	−0.022 (5)	−0.008 (5)
O6W	0.057 (6)	0.080 (8)	0.076 (7)	0.034 (6)	0.009 (5)	−0.001 (6)
O7	0.036 (4)	0.040 (4)	0.052 (5)	0.021 (3)	0.000 (3)	0.005 (3)
O7W	0.094 (10)	0.080 (9)	0.085 (9)	0.043 (8)	0.005 (7)	0.008 (7)
O8	0.050 (5)	0.058 (6)	0.052 (5)	0.031 (4)	−0.006 (4)	0.004 (4)
O9	0.039 (4)	0.042 (4)	0.050 (5)	0.017 (4)	0.000 (3)	−0.004 (3)
O10	0.062 (6)	0.061 (6)	0.049 (5)	0.037 (5)	−0.002 (4)	0.004 (4)
O11	0.047 (5)	0.060 (5)	0.043 (4)	0.033 (4)	0.004 (4)	0.003 (4)
O12	0.099 (9)	0.054 (6)	0.083 (8)	0.041 (7)	0.017 (7)	−0.003 (6)
O13	0.042 (4)	0.046 (5)	0.051 (5)	0.024 (4)	0.000 (4)	0.004 (4)
O14	0.045 (5)	0.056 (5)	0.042 (4)	0.026 (4)	0.000 (3)	−0.001 (4)
O15	0.044 (4)	0.040 (4)	0.043 (4)	0.020 (4)	0.003 (3)	0.000 (3)
O16	0.046 (5)	0.053 (6)	0.073 (6)	0.016 (4)	−0.001 (5)	0.010 (5)
O17	0.045 (5)	0.044 (5)	0.045 (4)	0.019 (4)	−0.001 (3)	−0.003 (4)
O18	0.051 (5)	0.052 (5)	0.061 (6)	0.024 (4)	0.006 (4)	0.004 (4)
O19	0.032 (4)	0.042 (4)	0.052 (5)	0.015 (3)	0.000 (3)	−0.003 (4)
O20	0.058 (6)	0.073 (6)	0.049 (5)	0.044 (5)	0.001 (4)	−0.007 (4)
O21	0.042 (4)	0.045 (5)	0.055 (5)	0.019 (4)	0.000 (4)	−0.002 (4)
O22	0.067 (6)	0.047 (5)	0.068 (6)	0.033 (5)	−0.002 (5)	−0.002 (4)
O23	0.044 (5)	0.042 (4)	0.052 (5)	0.025 (4)	−0.003 (4)	−0.001 (4)
O24	0.046 (5)	0.062 (6)	0.038 (4)	0.028 (4)	−0.001 (3)	0.001 (4)
O25	0.064 (6)	0.048 (5)	0.042 (4)	0.027 (4)	−0.005 (4)	−0.008 (4)
O26	0.048 (5)	0.039 (4)	0.051 (5)	0.016 (4)	0.001 (4)	−0.003 (4)
O27	0.061 (6)	0.059 (6)	0.050 (5)	0.034 (5)	−0.005 (4)	−0.005 (4)
O28	0.063 (6)	0.048 (5)	0.047 (5)	0.032 (4)	−0.002 (4)	0.008 (4)

### Geometric parameters (Å, º)

**Table:**

W1—O12	1.732 (12)	Cs5—O25^vi^	3.525 (9)
W1—O6	1.764 (11)	Cs6—O4	3.418 (11)
W1—O26	1.908 (9)	Cs6—O4^vii^	3.418 (10)
W1—O25	1.919 (10)	Cs6—O4^viii^	3.418 (10)
W1—O5	2.008 (9)	Cs6—O6^vii^	3.705 (13)
W2—O4	1.765 (10)	Cs6—O6^viii^	3.705 (14)
W2—O18	1.768 (11)	Cs6—O6	3.704 (14)
W2—O9	1.853 (9)	Cs6—O26^viii^	3.192 (10)
W2—O26	2.007 (9)	Cs6—O26^vii^	3.192 (10)
W2—O5	2.102 (9)	Cs6—O26	3.192 (10)
W2—O21^i^	2.287 (9)	Cs7—O2W^vii^	3.555 (19)
W3—O2	1.771 (10)	Cs7—O4	3.064 (10)
W3—O10	1.774 (9)	Cs7—O4W	3.414 (14)
W3—O15	1.817 (9)	Cs7—O12^vii^	3.198 (14)
W3—O25	2.046 (10)	Cs7—O13^ix^	3.772 (9)
W3—O5	2.092 (9)	Cs7—O16	3.279 (11)
W3—O24^i^	2.171 (9)	Cs7—O21^ix^	3.405 (10)
W4—O17	1.795 (9)	Cs7—O22^ix^	3.020 (12)
W4—O23	1.796 (9)	Cs7—O26^vii^	2.991 (10)
W4—O19	1.811 (8)	Cs8—O6W	3.517 (13)
W4—O9	2.031 (9)	Cs8—O6W^ix^	3.516 (13)
W4—O7^i^	2.078 (8)	Cs8—O6W^x^	3.516 (13)
W4—O15	2.124 (8)	Cs8—O11	3.149 (10)
W5—O16	1.747 (11)	Cs8—O11^x^	3.149 (10)
W5—O20	1.765 (9)	Cs8—O11^ix^	3.149 (10)
W5—O14	1.900 (9)	Cs8—O20	3.222 (11)
W5—O11	1.924 (9)	Cs8—O20^x^	3.222 (11)
W5—O1	2.007 (8)	Cs8—O20^ix^	3.222 (11)
W6—O27	1.746 (10)	Cs8—O22^ix^	3.451 (12)
W6—O28	1.778 (9)	Cs8—O22	3.451 (11)
W6—O3	1.846 (8)	Cs8—O22^x^	3.450 (11)
W6—O14	2.028 (9)	Cs9—O1	3.462 (8)
W6—O1	2.089 (8)	Cs9—O2W^ii^	3.442 (18)
W6—O17	2.212 (9)	Cs9—O14^xi^	3.048 (9)
W7—O22	1.750 (10)	Cs9—O16^x^	3.224 (12)
W7—O8	1.767 (10)	Cs9—O20^xi^	3.239 (10)
W7—O13	1.847 (9)	Cs9—O20^x^	3.078 (11)
W7—O11	2.003 (9)	Cs9—O20	3.577 (12)
W7—O1	2.088 (8)	Cs9—O22	3.339 (11)
W7—O23	2.293 (9)	Cs9—O27	3.009 (10)
W8—O21	1.784 (9)	Na1—O3W	2.409 (13)
W8—O24	1.805 (9)	Na1—O4W	2.346 (16)
W8—O7	1.829 (8)	Na1—O5W	2.421 (16)
W8—O13	2.011 (9)	Na1—O6W	2.356 (14)
W8—O3	2.066 (8)	Na1—O7W	2.375 (16)
W8—O19	2.121 (8)	Na1—O8	2.540 (12)
Cs1—O19^ii^	3.139 (9)	Na2—O6W^x^	2.380 (16)
Cs1—O19	3.139 (9)	Na2—O6W^ix^	2.380 (16)
Cs1—O19^i^	3.139 (9)	Na2—O6W	2.380 (16)
Cs1—O2^ii^	3.184 (10)	Na2—O7W^x^	2.508 (19)
Cs1—O2	3.184 (11)	Na2—O7W^ix^	2.508 (19)
Cs1—O2^i^	3.184 (10)	Na2—O7W	2.508 (19)
Cs1—O15^i^	3.426 (9)	Na3—O1W^iv^	2.77 (3)
Cs1—O15^ii^	3.426 (9)	Na3—O2W	3.81 (3)
Cs1—O15	3.426 (9)	Na3—O18	2.33 (2)
Cs2—O7^i^	3.168 (8)	O1W—O2W^iv^	3.44 (3)
Cs2—O7^ii^	3.168 (8)	O1W—O2W	4.02 (3)
Cs2—O7	3.168 (8)	O1W—O3^i^	3.69 (3)
Cs2—O28^i^	3.382 (10)	O1W—O18^iv^	3.86 (3)
Cs2—O28	3.382 (10)	O1W—O27^i^	3.53 (3)
Cs2—O28^ii^	3.382 (10)	O1W—O28	2.72 (3)
Cs3—O2^ii^	3.413 (10)	O1W—O28^i^	2.97 (3)
Cs2—O19^i^	3.514 (9)	O2—O3W^xii^	3.550 (16)
Cs2—O19^ii^	3.514 (9)	O2—O5W^xii^	3.238 (17)
Cs2—O19	3.514 (9)	O2—O7W^xii^	2.886 (19)
Cs1—O7^ii^	3.587 (9)	O2—O7W^iii^	3.342 (18)
Cs1—O7^i^	3.587 (9)	O2W—O3^i^	3.134 (17)
Cs1—O7	3.587 (9)	O2W—O18	3.023 (19)
Cs2—O3^i^	3.657 (9)	O2W—O21^i^	2.927 (17)
Cs2—O3^ii^	3.657 (9)	O2W—O27^i^	2.867 (19)
Cs2—O3	3.657 (9)	O3W—O4W	3.504 (18)
Cs3—O3W^iii^	3.202 (11)	O3W—O5W^xii^	4.146 (18)
Cs3—O3W	3.303 (11)	O3W—O5W	3.59 (2)
Cs3—O8	3.068 (10)	O3W—O7W	3.275 (19)
Cs3—O10	3.702 (11)	O3W—O10	2.690 (15)
Cs3—O13	3.274 (9)	O3W—O25^vi^	2.739 (15)
Cs3—O15	3.179 (9)	O4—O4W	2.857 (19)
Cs3—O19	3.545 (9)	O4W—O5W	3.52 (2)
Cs3—O23	3.288 (9)	O4W—O6W	3.18 (2)
Cs3—O24	2.943 (9)	O4W—O8	3.403 (18)
Cs3—O25^ii^	3.537 (11)	O4W—O9	3.292 (17)
Cs4—O1W^iv^	3.09 (3)	O4W—O11	3.331 (17)
Cs4—O1W	3.38 (2)	O4W—O12^vii^	3.842 (19)
Cs4—O2W	3.47 (2)	O4W—O23	2.775 (17)
Cs4—O2W^iv^	3.578 (17)	O5W—O6W	3.074 (19)
Cs4—O9	3.431 (9)	O5W—O7W	3.50 (2)
Cs4—O14	3.053 (10)	O5W—O7W^ix^	3.07 (2)
Cs4—O16	3.128 (11)	O5W—O8^ix^	3.633 (19)
Cs4—O17	3.171 (9)	O5W—O10^vi^	2.765 (16)
Cs4—O18	3.148 (11)	O5W—O12^vii^	2.745 (18)
Cs4—O27^v^	3.246 (10)	O6W—O6W^ix^	3.37 (2)
Cs5—O3W	3.386 (12)	O6W—O6W^x^	3.37 (2)
Cs5—O4	3.170 (11)	O6W—O7W	3.471 (19)
Cs5—O4W	3.275 (14)	O6W—O8	2.935 (15)
Cs5—O5	3.511 (9)	O6W—O8^ix^	2.991 (15)
Cs5—O5W	3.737 (19)	O6W—O11	2.989 (16)
Cs5—O6	3.469 (15)	O6W—O22^ix^	2.787 (16)
Cs5—O6^vi^	3.127 (12)	O7W—O7W^x^	3.26 (3)
Cs5—O6^vii^	3.193 (13)	O7W—O7W^ix^	3.26 (3)
Cs5—O10	3.302 (11)	O7W—O8	3.259 (18)
Cs5—O12^vii^	3.529 (15)		
			
O5—W1—O5W^viii^	149.4 (4)	Na3^iv^—O1W—Cs4^iv^	84.8 (8)
O6—W1—O5	114.5 (5)	Na3^iv^—O1W—Cs4	137.7 (10)
O6—W1—O5W^viii^	92.3 (5)	Na3^iv^—O1W—O2W^iv^	74.7 (8)
O6—W1—O25	101.9 (5)	Na3^iv^—O1W—O2W	140.9 (10)
O6—W1—O26	100.7 (5)	Na3^iv^—O1W—O3^i^	136.8 (10)
O12—W1—O5	137.4 (6)	Na3^iv^—O1W—O18^iv^	36.7 (6)
O12—W1—O5W^viii^	23.3 (5)	Na3^iv^—O1W—O27^i^	106.9 (9)
O12—W1—O6	108.0 (7)	Na3^iv^—O1W—O28^i^	88.1 (9)
O12—W1—O25	98.8 (6)	O2W^iv^—O1W—W6^i^	148.6 (8)
O12—W1—O26	95.7 (5)	O2W^iv^—O1W—Cs2	143.1 (8)
O25—W1—O5	75.2 (4)	O2W^iv^—O1W—O2W	97.9 (7)
O25—W1—O5W^viii^	85.4 (4)	O2W^iv^—O1W—O3^i^	144.1 (8)
O26—W1—O5	74.7 (4)	O2W^iv^—O1W—O18^iv^	48.5 (4)
O26—W1—O5W^viii^	116.3 (3)	O2W^iv^—O1W—O27^i^	121.4 (8)
O26—W1—O25	147.8 (4)	O3^i^—O1W—W6^i^	28.6 (2)
O4—W2—O2W	142.9 (4)	O3^i^—O1W—Cs2	57.2 (4)
O4—W2—O4W	44.1 (4)	O3^i^—O1W—O2W	47.7 (4)
O4—W2—O5	92.9 (4)	O3^i^—O1W—O18^iv^	142.6 (7)
O4—W2—O9	100.2 (5)	O18^iv^—O1W—Cs2	142.8 (7)
O4—W2—O18	102.8 (5)	O18^iv^—O1W—O2W	111.0 (7)
O4—W2—O21^i^	173.6 (4)	O27^i^—O1W—W6^i^	27.3 (2)
O4—W2—O26	96.9 (5)	O27^i^—O1W—Cs2	94.1 (6)
O4W—W2—O2W	143.7 (4)	O27^i^—O1W—O2W	44.0 (4)
O5—W2—O2W	123.4 (3)	O27^i^—O1W—O3^i^	44.8 (4)
O5—W2—O4W	76.6 (3)	O27^i^—O1W—O18^iv^	97.9 (6)
O5—W2—O21^i^	80.9 (3)	O28—O1W—W6^i^	124.2 (8)
O9—W2—O2W	90.5 (4)	O28^i^—O1W—W6^i^	27.2 (3)
O9—W2—O4W	58.7 (4)	O28—O1W—Cs2	57.7 (5)
O9—W2—O5	85.9 (4)	O28^i^—O1W—Cs2	56.6 (5)
O9—W2—O21^i^	81.0 (4)	O28—O1W—Cs4	80.1 (6)
O9—W2—O26	151.6 (4)	O28^i^—O1W—Cs4	133.4 (9)
O18—W2—O2W	40.1 (4)	O28—O1W—Cs4^iv^	148.9 (10)
O18—W2—O4W	123.9 (4)	O28^i^—O1W—Cs4^iv^	99.6 (7)
O18—W2—O5	159.5 (4)	O28—O1W—Na3^iv^	92.6 (9)
O18—W2—O9	103.8 (5)	O28^i^—O1W—O2W	85.4 (7)
O18—W2—O21^i^	82.9 (4)	O28^i^—O1W—O2W^iv^	156.7 (9)
O18—W2—O26	94.3 (4)	O28—O1W—O2W^iv^	85.5 (7)
O21^i^—W2—O2W	43.0 (3)	O28—O1W—O2W	125.5 (9)
O21^i^—W2—O4W	134.6 (3)	O28—O1W—O3^i^	105.4 (8)
O26—W2—O2W	89.4 (4)	O28^i^—O1W—O3^i^	48.8 (4)
O26—W2—O4W	126.8 (4)	O28—O1W—O18^iv^	111.3 (8)
O26—W2—O5	70.6 (4)	O28^i^—O1W—O18^iv^	108.7 (7)
O26—W2—O21^i^	79.7 (4)	O28^i^—O1W—O27^i^	48.4 (4)
O2—W3—O3W^xii^	58.7 (4)	O28—O1W—O27^i^	149.7 (10)
O2—W3—O5	158.4 (4)	O28—O1W—O28^i^	111.4 (9)
O2—W3—O5W^xii^	61.3 (4)	W3—O2—Cs1	105.1 (4)
O2—W3—O10	101.2 (5)	W3—O2—Cs3^i^	100.9 (4)
O2—W3—O15	103.5 (4)	W3—O2—O3W^xii^	96.1 (4)
O2—W3—O24^i^	85.8 (4)	W3—O2—O5W^xii^	90.0 (4)
O2—W3—O25	93.3 (5)	W3—O2—O7W^iii^	119.7 (5)
O5—W3—O3W^xii^	106.2 (3)	W3—O2—O7W^xii^	153.4 (6)
O5—W3—O5W^xii^	124.9 (3)	Cs1—O2—Cs3^i^	88.1 (2)
O5—W3—O24^i^	77.1 (3)	Cs1—O2—O3W^xii^	140.3 (4)
O5W^xii^—W3—O3W^xii^	54.2 (3)	Cs1—O2—O5W^xii^	147.2 (5)
O10—W3—O3W^xii^	93.2 (4)	Cs1—O2—O7W^iii^	92.0 (4)
O10—W3—O5	94.7 (4)	Cs3^i^—O2—O3W^xii^	54.7 (2)
O10—W3—O5W^xii^	45.9 (4)	O5W^xii^—O2—Cs3^i^	118.0 (4)
O10—W3—O15	100.4 (4)	O5W^xii^—O2—O3W^xii^	63.6 (4)
O10—W3—O24^i^	170.7 (5)	O5W^xii^—O2—O7W^iii^	55.5 (5)
O10—W3—O25	94.2 (4)	O7W^xii^—O2—Cs1	101.2 (4)
O15—W3—O3W^xii^	159.7 (3)	O7W^xii^—O2—Cs3^i^	75.9 (4)
O15—W3—O5	87.8 (4)	O7W^iii^—O2—Cs3^i^	137.7 (4)
O15—W3—O5W^xii^	128.9 (4)	O7W^iii^—O2—O3W^xii^	106.3 (4)
O15—W3—O24^i^	83.8 (4)	O7W^xii^—O2—O3W^xii^	60.1 (4)
O15—W3—O25	155.0 (4)	O7W^xii^—O2—O5W^xii^	69.5 (4)
O24^i^—W3—O3W^xii^	85.1 (3)	O7W^xii^—O2—O7W^iii^	62.6 (6)
O24^i^—W3—O5W^xii^	136.4 (3)	W6^i^—O2W—W8^i^	59.0 (2)
O25—W3—W1	34.0 (3)	W6^i^—O2W—Na3	170.9 (6)
O25—W3—O3W^xii^	35.5 (3)	Cs4—O2W—W6^i^	106.2 (4)
O25—W3—O5	70.7 (4)	Cs4^iv^—O2W—W6^i^	78.9 (3)
O25—W3—O5W^xii^	75.5 (4)	Cs4—O2W—W8^i^	87.6 (3)
O25—W3—O24^i^	79.1 (4)	Cs4^iv^—O2W—W8^i^	126.7 (5)
O7^i^—W4—O4W	133.5 (3)	Cs4—O2W—Cs4^iv^	73.5 (4)
O7^i^—W4—O15	80.7 (3)	Cs4—O2W—Cs7^viii^	128.4 (4)
O9—W4—O4W	56.3 (3)	Cs4—O2W—Na3	65.7 (5)
O9—W4—O7^i^	83.1 (4)	Cs4^iv^—O2W—Na3	102.0 (5)
O9—W4—O15	84.9 (3)	Cs7^viii^—O2W—W6^i^	105.9 (5)
O15—W4—O4W	74.7 (3)	Cs7^viii^—O2W—W8^i^	75.8 (3)
O17—W4—O4W	112.3 (4)	Cs7^viii^—O2W—Cs4^iv^	152.3 (5)
O17—W4—O7^i^	89.0 (4)	Cs7^viii^—O2W—Na3	77.6 (5)
O17—W4—O9	92.6 (4)	Cs9^i^—O2W—W6^i^	69.5 (3)
O17—W4—O15	169.6 (4)	Cs9^i^—O2W—W8^i^	104.7 (4)
O17—W4—O19	95.0 (4)	Cs9^i^—O2W—Cs4^iv^	87.3 (3)
O17—W4—O23	98.3 (4)	Cs9^i^—O2W—Cs4	160.8 (5)
O19—W4—O4W	127.7 (4)	Cs9^i^—O2W—Cs7^viii^	69.8 (4)
O19—W4—O7^i^	88.2 (4)	Cs9^i^—O2W—Na3	119.6 (6)
O19—W4—O9	168.3 (4)	Cs9^i^—O2W—O1W^iv^	115.8 (6)
O19—W4—O15	86.0 (4)	Na3—O2W—W8^i^	114.8 (5)
O23—W4—O4W	38.3 (4)	O1W^iv^—O2W—W6^i^	134.8 (7)
O23—W4—O7^i^	171.0 (4)	O1W^iv^—O2W—W8^i^	139.5 (7)
O23—W4—O9	91.2 (4)	O1W^iv^—O2W—Cs4	53.2 (5)
O23—W4—O15	91.9 (4)	O1W^iv^—O2W—Cs4^iv^	57.6 (5)
O23—W4—O19	96.5 (4)	O1W^iv^—O2W—Cs7^viii^	118.2 (6)
O14—W5—O1	74.6 (4)	O1W^iv^—O2W—Na3	44.6 (6)
O14—W5—O11	147.9 (4)	O3^i^—O2W—W6^i^	28.9 (2)
O16—W5—O1	139.8 (5)	O3^i^—O2W—W8^i^	31.4 (2)
O16—W5—O11	97.6 (5)	O3^i^—O2W—Cs4	92.3 (5)
O16—W5—O14	98.0 (5)	O3^i^—O2W—Cs4^iv^	98.8 (5)
O16—W5—O20	106.6 (5)	O3^i^—O2W—Cs7^viii^	97.0 (5)
O20—W5—O1	113.6 (4)	O3^i^—O2W—Cs9^i^	91.0 (5)
O20—W5—O11	100.6 (4)	O3^i^—O2W—Na3	143.4 (6)
O20—W5—O14	101.5 (4)	O3^i^—O2W—O1W^iv^	140.9 (8)
O1—W6—O1W^ii^	149.5 (5)	O18—O2W—W6^i^	134.9 (5)
O1—W6—O2W^ii^	86.2 (4)	O18—O2W—W8^i^	77.5 (4)
O1—W6—O17	78.2 (3)	O18—O2W—Cs4^iv^	125.0 (6)
O3—W6—O1	85.1 (4)	O18—O2W—Cs4	57.5 (4)
O3—W6—O1W^ii^	72.7 (5)	O18—O2W—Cs7^viii^	71.2 (4)
O3—W6—O2W^ii^	55.0 (4)	O18—O2W—Cs9^i^	138.9 (7)
O3—W6—O14	152.2 (4)	O18—O2W—Na3	37.6 (4)
O3—W6—O17	85.4 (4)	O18—O2W—O1W^iv^	72.9 (6)
O14—W6—O1	70.3 (3)	O18—O2W—O3^i^	106.1 (5)
O14—W6—O1W^ii^	134.9 (5)	O21^i^—O2W—W6^i^	83.8 (4)
O14—W6—O2W^ii^	132.5 (4)	O21^i^—O2W—W8^i^	24.9 (2)
O14—W6—O17	77.2 (4)	O21^i^—O2W—Cs4^iv^	144.6 (6)
O17—W6—O1W^ii^	119.4 (5)	O21^i^—O2W—Cs4	82.2 (4)
O17—W6—O2W^ii^	138.7 (3)	O21^i^—O2W—Cs7^viii^	62.5 (4)
O27—W6—O1	96.4 (4)	O21^i^—O2W—Cs9^i^	115.1 (6)
O27—W6—O1W^ii^	68.2 (5)	O21^i^—O2W—Na3	90.7 (5)
O27—W6—O2W^ii^	45.6 (4)	O21^i^—O2W—O1W^iv^	124.4 (7)
O27—W6—O3	100.2 (4)	O21^i^—O2W—O3^i^	56.2 (4)
O27—W6—O14	95.4 (4)	O21^i^—O2W—O18	54.2 (4)
O27—W6—O17	171.9 (4)	O27^i^—O2W—W6^i^	25.8 (2)
O27—W6—O28	100.9 (5)	O27^i^—O2W—W8^i^	84.7 (4)
O28—W6—O1	159.4 (4)	O27^i^—O2W—Cs4	111.8 (6)
O28—W6—O1W^ii^	50.0 (5)	O27^i^—O2W—Cs4^iv^	59.3 (4)
O28—W6—O2W^ii^	114.0 (5)	O27^i^—O2W—Cs7^viii^	114.7 (6)
O28—W6—O3	102.6 (4)	O27^i^—O2W—Cs9^i^	56.1 (4)
O28—W6—O14	96.8 (4)	O27^i^—O2W—Na3	159.6 (7)
O28—W6—O17	83.4 (4)	O27^i^—O2W—O1W^iv^	116.6 (7)
O1—W7—O4W	102.6 (3)	O27^i^—O2W—O3^i^	54.4 (4)
O1—W7—O6W	121.9 (3)	O27^i^—O2W—O18	159.3 (7)
O1—W7—O6W^x^	136.8 (3)	O27^i^—O2W—O21^i^	109.4 (6)
O1—W7—O23	79.5 (3)	W6—O3—W8	154.6 (5)
O6W—W7—O4W	47.5 (3)	W6—O3—Cs2	96.1 (3)
O6W^x^—W7—O4W	97.9 (3)	W6—O3—O1W^ii^	78.8 (5)
O6W^x^—W7—O6W	53.5 (4)	W6—O3—O2W^ii^	96.1 (4)
O8—W7—O1	159.7 (4)	W8—O3—Cs2	92.0 (3)
O8—W7—O4W	57.2 (4)	W8—O3—O1W^ii^	126.2 (5)
O8—W7—O6W^x^	55.1 (4)	W8—O3—O2W^ii^	96.5 (4)
O8—W7—O6W	46.6 (4)	Cs2—O3—O1W^ii^	64.9 (4)
O8—W7—O11	94.3 (4)	O2W^ii^—O3—Cs2	131.4 (5)
O8—W7—O13	102.6 (4)	O2W^ii^—O3—O1W^ii^	71.8 (6)
O8—W7—O23	83.6 (4)	W3^vi^—O3W—O5W^xii^	101.4 (3)
O11—W7—O1	71.9 (3)	Cs3^iii^—O3W—W3^vi^	67.2 (2)
O11—W7—O4W	55.7 (3)	Cs3—O3W—W3^vi^	151.4 (3)
O11—W7—O6W	50.0 (3)	Cs3^iii^—O3W—Cs3	90.1 (3)
O11—W7—O6W^x^	89.8 (3)	Cs3—O3W—Cs5	124.5 (3)
O11—W7—O23	79.5 (4)	Cs3^iii^—O3W—Cs5	139.8 (4)
O13—W7—O1	85.7 (4)	Cs3^iii^—O3W—O2^vi^	60.5 (3)
O13—W7—O4W	117.2 (4)	Cs3—O3W—O2^vi^	128.0 (4)
O13—W7—O6W^x^	117.6 (4)	Cs3^iii^—O3W—O4W	160.9 (4)
O13—W7—O6W	148.9 (3)	Cs3—O3W—O4W	81.6 (3)
O13—W7—O11	152.5 (4)	Cs3—O3W—O5W^xii^	87.7 (3)
O13—W7—O23	81.1 (4)	Cs3^iii^—O3W—O5W^xii^	73.6 (3)
O22—W7—O1	94.3 (4)	Cs3—O3W—O5W	125.6 (4)
O22—W7—O4W	139.3 (4)	Cs3^iii^—O3W—O5W	114.2 (4)
O22—W7—O6W^x^	48.3 (4)	Cs3^iii^—O3W—O7W	74.0 (4)
O22—W7—O6W	92.2 (4)	Cs5—O3W—W3^vi^	83.4 (2)
O22—W7—O8	102.2 (5)	Cs5—O3W—O2^vi^	102.6 (3)
O22—W7—O11	96.7 (5)	Cs5—O3W—O4W	56.7 (3)
O22—W7—O13	100.6 (5)	Cs5—O3W—O5W^xii^	86.7 (3)
O22—W7—O23	173.4 (4)	Cs5—O3W—O5W	64.8 (3)
O23—W7—O4W	41.9 (3)	Na1—O3W—W3^vi^	93.1 (4)
O23—W7—O6W	89.5 (3)	Na1—O3W—Cs3^iii^	120.4 (4)
O23—W7—O6W^x^	136.5 (3)	Na1—O3W—Cs3	83.4 (3)
O3—W8—O2W^ii^	52.2 (3)	Na1—O3W—Cs5	86.8 (4)
O3—W8—O19	79.1 (3)	Na1—O3W—O2^vi^	78.1 (4)
O7—W8—O2W^ii^	97.9 (4)	Na1—O3W—O4W	41.9 (3)
O7—W8—O3	89.4 (4)	Na1—O3W—O5W^xii^	163.2 (5)
O7—W8—O13	170.0 (4)	Na1—O3W—O5W	42.2 (3)
O7—W8—O19	87.3 (4)	Na1—O3W—O7W	46.4 (4)
O13—W8—O2W^ii^	84.4 (4)	Na1—O3W—O10	122.4 (5)
O13—W8—O3	84.3 (4)	Na1—O3W—O25^vi^	112.7 (5)
O13—W8—O19	83.8 (4)	O2^vi^—O3W—W3^vi^	25.22 (17)
O19—W8—O2W^ii^	130.7 (3)	O2^vi^—O3W—O5W^xii^	118.4 (4)
O21—W8—O2W^ii^	43.7 (4)	O2^vi^—O3W—O5W	54.0 (3)
O21—W8—O3	95.8 (4)	O4W—O3W—W3^vi^	114.3 (4)
O21—W8—O7	97.0 (4)	O4W—O3W—O2^vi^	112.0 (4)
O21—W8—O13	91.5 (4)	O4W—O3W—O5W^xii^	122.8 (4)
O21—W8—O19	173.3 (4)	O4W—O3W—O5W	59.4 (4)
O21—W8—O24	97.1 (4)	O5W—O3W—W3^vi^	56.6 (3)
O24—W8—O2W^ii^	140.0 (4)	O5W—O3W—O5W^xii^	144.3 (5)
O24—W8—O3	165.9 (4)	O7W—O3W—W3^vi^	74.6 (4)
O24—W8—O7	94.7 (4)	O7W—O3W—Cs3	82.8 (4)
O24—W8—O13	89.6 (4)	O7W—O3W—Cs5	125.2 (4)
O24—W8—O19	87.6 (4)	O7W—O3W—O2^vi^	49.8 (4)
O2^i^—Cs1—O2	81.6 (3)	O7W—O3W—O4W	87.9 (4)
O2^ii^—Cs1—O2	81.6 (3)	O7W—O3W—O5W	61.2 (4)
O2^i^—Cs1—O2^ii^	81.6 (3)	O7W—O3W—O5W^xii^	146.1 (5)
O2^ii^—Cs1—O7	83.2 (2)	O10—O3W—W3^vi^	128.3 (4)
O2^i^—Cs1—O7^ii^	83.2 (2)	O10—O3W—Cs3	75.5 (3)
O2^ii^—Cs1—O7^i^	129.9 (2)	O10—O3W—Cs3^iii^	112.7 (4)
O2—Cs1—O7	142.2 (2)	O10—O3W—Cs5	64.7 (3)
O2^i^—Cs1—O7^i^	142.2 (2)	O10—O3W—O2^vi^	153.0 (5)
O2—Cs1—O7^ii^	129.9 (2)	O10—O3W—O4W	81.9 (4)
O2—Cs1—O7^i^	83.2 (2)	O10—O3W—O5W^xii^	41.2 (3)
O2^i^—Cs1—O7	129.9 (2)	O10—O3W—O5W	127.6 (5)
O2^ii^—Cs1—O7^ii^	142.2 (2)	O10—O3W—O7W	157.1 (5)
O2^i^—Cs1—O15^i^	50.3 (2)	O10—O3W—O25^vi^	102.7 (5)
O2—Cs1—O15	50.3 (2)	O25^vi^—O3W—W3^vi^	25.7 (2)
O2^i^—Cs1—O15	131.9 (2)	O25^vi^—O3W—Cs3^iii^	72.6 (3)
O2^ii^—Cs1—O15^i^	131.9 (2)	O25^vi^—O3W—Cs3	160.6 (5)
O2—Cs1—O15^ii^	131.9 (2)	O25^vi^—O3W—Cs5	69.4 (3)
O2—Cs1—O15^i^	89.9 (2)	O25^vi^—O3W—O2^vi^	50.5 (3)
O2^ii^—Cs1—O15	89.9 (2)	O25^vi^—O3W—O4W	117.6 (5)
O2^i^—Cs1—O15^ii^	89.9 (2)	O25^vi^—O3W—O5W^xii^	79.2 (4)
O2^ii^—Cs1—O15^ii^	50.3 (2)	O25^vi^—O3W—O5W	71.2 (4)
O7—Cs1—O7^i^	80.5 (2)	O25^vi^—O3W—O7W	100.3 (5)
O7^ii^—Cs1—O7^i^	80.5 (2)	W2—O4—Cs5	119.7 (5)
O7^ii^—Cs1—O7	80.5 (2)	W2—O4—Cs6	100.8 (4)
O15^ii^—Cs1—O7^ii^	95.40 (19)	W2—O4—Cs7	132.9 (5)
O15—Cs1—O7	95.40 (19)	W2—O4—O4W	110.5 (5)
O15—Cs1—O7^ii^	125.41 (19)	Cs5—O4—Cs6	85.5 (3)
O15^ii^—Cs1—O7^i^	125.41 (19)	Cs7—O4—Cs5	103.8 (3)
O15^i^—Cs1—O7^i^	95.40 (19)	Cs7—O4—Cs6	99.8 (3)
O15—Cs1—O7^i^	45.6 (2)	O4W—O4—Cs5	65.6 (4)
O15^i^—Cs1—O7	125.41 (19)	O4W—O4—Cs6	144.9 (5)
O15^i^—Cs1—O7^ii^	45.6 (2)	O4W—O4—Cs7	70.3 (4)
O15^ii^—Cs1—O7	45.6 (2)	Cs5—O4W—Cs7	94.3 (4)
O15^ii^—Cs1—O15	119.982 (7)	Cs5—O4W—O3W	59.8 (3)
O15^i^—Cs1—O15	119.983 (9)	Cs5—O4W—O5W	66.7 (4)
O15^i^—Cs1—O15^ii^	119.983 (8)	Cs5—O4W—O8	131.9 (5)
O19—Cs1—O2^ii^	88.4 (2)	Cs5—O4W—O9	98.3 (4)
O19—Cs1—O2	97.7 (2)	Cs5—O4W—O11	175.1 (5)
O19—Cs1—O2^i^	170.0 (2)	Cs5—O4W—O12^vii^	58.8 (3)
O19^ii^—Cs1—O2^i^	88.4 (2)	Cs7—O4W—O3W	146.6 (5)
O19^i^—Cs1—O2^i^	97.7 (2)	Cs7—O4W—O5W	90.1 (4)
O19^i^—Cs1—O2^ii^	170.0 (2)	Cs7—O4W—O12^vii^	51.9 (3)
O19^ii^—Cs1—O2^ii^	97.7 (2)	Na1—O4W—Cs5	90.4 (5)
O19^i^—Cs1—O2	88.4 (2)	Na1—O4W—Cs7	125.4 (5)
O19^ii^—Cs1—O2	170.0 (2)	Na1—O4W—O3W	43.2 (3)
O19—Cs1—O7^ii^	104.6 (2)	Na1—O4W—O4	151.8 (7)
O19^ii^—Cs1—O7	47.00 (19)	Na1—O4W—O5W	43.3 (4)
O19—Cs1—O7^i^	47.00 (19)	Na1—O4W—O6W	47.6 (4)
O19^ii^—Cs1—O7^ii^	47.41 (19)	Na1—O4W—O8	48.2 (3)
O19^i^—Cs1—O7	104.6 (2)	Na1—O4W—O9	144.0 (6)
O19^ii^—Cs1—O7^i^	104.6 (2)	Na1—O4W—O11	87.5 (5)
O19^i^—Cs1—O7^ii^	47.00 (19)	Na1—O4W—O12^vii^	86.7 (5)
O19^i^—Cs1—O7^i^	47.41 (19)	Na1—O4W—O23	96.1 (5)
O19—Cs1—O7	47.41 (19)	O3W—O4W—O5W	61.4 (4)
O19—Cs1—O15	48.2 (2)	O3W—O4W—O12^vii^	95.0 (4)
O19^ii^—Cs1—O15	139.7 (2)	O4—O4W—Cs5	61.8 (4)
O19—Cs1—O15^i^	139.7 (2)	O4—O4W—Cs7	57.7 (3)
O19^ii^—Cs1—O15^ii^	48.2 (2)	O4—O4W—O3W	116.1 (5)
O19^i^—Cs1—O15^i^	48.2 (2)	O4—O4W—O5W	114.3 (5)
O19^i^—Cs1—O15^ii^	139.7 (2)	O4—O4W—O6W	142.4 (6)
O19^i^—Cs1—O15	83.2 (2)	O4—O4W—O8	157.2 (6)
O19—Cs1—O15^ii^	83.2 (2)	O4—O4W—O9	53.1 (3)
O19^ii^—Cs1—O15^i^	83.2 (2)	O4—O4W—O11	119.8 (5)
O19^ii^—Cs1—O19^i^	92.3 (2)	O4—O4W—O12^vii^	75.2 (4)
O19^i^—Cs1—O19	92.3 (2)	O5W—O4W—O12^vii^	43.5 (3)
O19^ii^—Cs1—O19	92.3 (2)	O6W—O4W—Cs5	121.0 (5)
O3^i^—Cs2—O3	119.75 (2)	O6W—O4W—Cs7	85.2 (4)
O3^ii^—Cs2—O3	119.75 (2)	O6W—O4W—O3W	90.8 (5)
O3^ii^—Cs2—O3^i^	119.75 (2)	O6W—O4W—O5W	54.4 (4)
O7^ii^—Cs2—O3^ii^	46.8 (2)	O6W—O4W—O8	52.8 (4)
O7—Cs2—O3^i^	139.4 (2)	O6W—O4W—O9	140.5 (6)
O7^i^—Cs2—O3	81.1 (2)	O6W—O4W—O11	54.6 (4)
O7—Cs2—O3^ii^	81.1 (2)	O6W—O4W—O12^vii^	76.7 (4)
O7^ii^—Cs2—O3	139.4 (2)	O8—O4W—Cs7	127.7 (5)
O7^ii^—Cs2—O3^i^	81.1 (2)	O8—O4W—O3W	72.2 (4)
O7—Cs2—O3	46.77 (19)	O8—O4W—O5W	88.4 (4)
O7^i^—Cs2—O3^i^	46.8 (2)	O8—O4W—O12^vii^	126.7 (5)
O7^i^—Cs2—O3^ii^	139.4 (2)	O9—O4W—Cs7	88.9 (4)
O7^i^—Cs2—O7	94.0 (2)	O9—O4W—O3W	113.8 (5)
O7^ii^—Cs2—O7	94.0 (2)	O9—O4W—O5W	164.8 (6)
O7^ii^—Cs2—O7^i^	94.0 (2)	O9—O4W—O8	104.1 (4)
O7^i^—Cs2—O19	47.57 (19)	O9—O4W—O11	86.0 (4)
O7—Cs2—O19^i^	105.7 (2)	O9—O4W—O12^vii^	127.6 (5)
O7^ii^—Cs2—O19^ii^	47.97 (19)	O11—O4W—Cs7	83.3 (4)
O7^i^—Cs2—O19^i^	47.97 (19)	O11—O4W—O3W	120.7 (5)
O7^i^—Cs2—O19^ii^	105.7 (2)	O11—O4W—O5W	109.0 (5)
O7^ii^—Cs2—O19^i^	47.56 (19)	O11—O4W—O8	48.5 (3)
O7—Cs2—O19^ii^	47.56 (19)	O11—O4W—O12^vii^	116.6 (5)
O7^ii^—Cs2—O19	105.7 (2)	O23—O4W—Cs5	132.0 (5)
O7—Cs2—O19	47.97 (19)	O23—O4W—Cs7	118.8 (5)
O7—Cs2—O28	92.8 (2)	O23—O4W—O3W	94.6 (5)
O7^ii^—Cs2—O28	173.2 (2)	O23—O4W—O4	105.9 (5)
O7^i^—Cs2—O28	85.2 (2)	O23—O4W—O5W	139.0 (6)
O7—Cs2—O28^i^	173.2 (2)	O23—O4W—O6W	97.0 (5)
O7^i^—Cs2—O28^ii^	173.2 (2)	O23—O4W—O8	51.3 (3)
O7^i^—Cs2—O28^i^	92.8 (2)	O23—O4W—O9	52.9 (3)
O7—Cs2—O28^ii^	85.2 (2)	O23—O4W—O11	52.7 (3)
O7^ii^—Cs2—O28^i^	85.2 (2)	O23—O4W—O12^vii^	168.6 (6)
O7^ii^—Cs2—O28^ii^	92.8 (2)	W1—O5—W2	103.5 (4)
O19—Cs2—O3^ii^	123.27 (19)	W1—O5—W3	104.3 (4)
O19^i^—Cs2—O3^i^	43.58 (19)	W1—O5—Cs5	92.7 (3)
O19^i^—Cs2—O3^ii^	94.32 (18)	W1—O5—O4W	141.6 (4)
O19^i^—Cs2—O3	123.27 (19)	W2—O5—Cs5	97.6 (3)
O19^ii^—Cs2—O3^i^	123.27 (19)	W2—O5—O4W	72.1 (3)
O19^ii^—Cs2—O3	94.32 (18)	W3—O5—W2	146.3 (5)
O19^ii^—Cs2—O3^ii^	43.58 (19)	W3—O5—Cs5	99.8 (3)
O19—Cs2—O3	43.59 (19)	W3—O5—O4W	96.8 (3)
O19—Cs2—O3^i^	94.32 (18)	Cs5—O5—O4W	51.8 (2)
O19^i^—Cs2—O19^ii^	80.2 (2)	W3^vi^—O5W—Cs5	85.4 (3)
O19^ii^—Cs2—O19	80.2 (2)	Na1—O5W—W3^vi^	104.6 (5)
O19^i^—Cs2—O19	80.2 (2)	Na1—O5W—Cs5	78.9 (5)
O28—Cs2—O3^i^	93.5 (2)	Na1—O5W—O2^vi^	84.6 (5)
O28^ii^—Cs2—O3	93.5 (2)	Na1—O5W—O3W	42.0 (4)
O28^i^—Cs2—O3^i^	47.2 (2)	Na1—O5W—O4W	41.7 (4)
O28—Cs2—O3^ii^	135.1 (2)	Na1—O5W—O6W	49.0 (4)
O28—Cs2—O3	47.17 (19)	Na1—O5W—O7W	42.6 (4)
O28^i^—Cs2—O3^ii^	93.5 (2)	Na1—O5W—O7W^ix^	95.3 (6)
O28^ii^—Cs2—O3^i^	135.1 (2)	Na1—O5W—O8^ix^	100.9 (5)
O28^i^—Cs2—O3	135.1 (2)	Na1—O5W—O10^vi^	130.9 (6)
O28^ii^—Cs2—O3^ii^	47.18 (19)	Na1—O5W—O12^vii^	116.0 (7)
O28—Cs2—O19^ii^	138.7 (2)	O2^vi^—O5W—W3^vi^	28.7 (2)
O28^i^—Cs2—O19^ii^	129.7 (2)	O2^vi^—O5W—Cs5	101.6 (5)
O28^ii^—Cs2—O19^i^	138.7 (2)	O2^vi^—O5W—O3W	62.5 (4)
O28^ii^—Cs2—O19^ii^	78.7 (2)	O2^vi^—O5W—O4W	119.9 (6)
O28^ii^—Cs2—O19	129.7 (2)	O2^vi^—O5W—O7W	50.5 (4)
O28^i^—Cs2—O19^i^	78.7 (2)	O2^vi^—O5W—O8^ix^	121.4 (6)
O28—Cs2—O19^i^	129.7 (2)	O3W—O5W—W3^vi^	69.2 (3)
O28^i^—Cs2—O19	138.7 (2)	O3W—O5W—Cs5	55.0 (3)
O28—Cs2—O19	78.7 (2)	O3W—O5W—O8^ix^	142.6 (5)
O28^i^—Cs2—O28	88.2 (2)	O4W—O5W—W3^vi^	126.1 (5)
O28^ii^—Cs2—O28	88.2 (2)	O4W—O5W—Cs5	53.6 (3)
O28^i^—Cs2—O28^ii^	88.2 (2)	O4W—O5W—O3W	59.1 (4)
O2^ii^—Cs3—O10	99.2 (2)	O4W—O5W—O8^ix^	97.9 (4)
O2^ii^—Cs3—O19	78.6 (2)	O6W—O5W—W3^vi^	141.3 (5)
O2^ii^—Cs3—O25^ii^	47.2 (2)	O6W—O5W—Cs5	110.8 (5)
O3W—Cs3—O2^ii^	129.7 (3)	O6W—O5W—O2^vi^	112.7 (5)
O3W^iii^—Cs3—O2^ii^	64.8 (3)	O6W—O5W—O3W	91.0 (5)
O3W^iii^—Cs3—O3W	89.9 (3)	O6W—O5W—O4W	57.2 (4)
O3W—Cs3—O10	44.7 (2)	O6W—O5W—O7W	63.3 (4)
O3W^iii^—Cs3—O10	106.3 (3)	O6W—O5W—O8^ix^	52.2 (4)
O3W^iii^—Cs3—O13	123.6 (3)	O7W^ix^—O5W—W3^vi^	82.8 (4)
O3W^iii^—Cs3—O19	140.6 (2)	O7W—O5W—W3^vi^	78.2 (4)
O3W—Cs3—O19	126.4 (2)	O7W^ix^—O5W—Cs5	165.1 (5)
O3W^iii^—Cs3—O23	166.6 (3)	O7W—O5W—Cs5	109.5 (5)
O3W^iii^—Cs3—O25^ii^	47.6 (2)	O7W^ix^—O5W—O2^vi^	64.0 (4)
O3W—Cs3—O25^ii^	136.8 (2)	O7W^ix^—O5W—O3W	111.8 (5)
O8—Cs3—O2^ii^	150.3 (3)	O7W—O5W—O3W	55.0 (4)
O8—Cs3—O3W	79.3 (3)	O7W^ix^—O5W—O4W	128.9 (6)
O8—Cs3—O3W^iii^	116.0 (3)	O7W—O5W—O4W	84.3 (5)
O8—Cs3—O10	108.2 (3)	O7W^ix^—O5W—O6W	74.0 (5)
O8—Cs3—O13	52.7 (2)	O7W^ix^—O5W—O7W	59.1 (6)
O8—Cs3—O15	100.4 (2)	O7W^ix^—O5W—O8^ix^	57.5 (4)
O8—Cs3—O19	88.4 (2)	O7W—O5W—O8^ix^	96.8 (5)
O8—Cs3—O23	50.8 (2)	O8^ix^—O5W—W3^vi^	134.3 (5)
O8—Cs3—O25^ii^	109.3 (3)	O8^ix^—O5W—Cs5	136.9 (4)
O13—Cs3—O2^ii^	100.6 (2)	O10^vi^—O5W—W3^vi^	27.4 (2)
O13—Cs3—O3W	129.0 (3)	O10^vi^—O5W—Cs5	85.2 (5)
O13—Cs3—O10	130.2 (2)	O10^vi^—O5W—O2^vi^	53.6 (4)
O13—Cs3—O19	47.6 (2)	O10^vi^—O5W—O3W	91.6 (5)
O13—Cs3—O23	48.8 (2)	O10^vi^—O5W—O4W	137.7 (7)
O13—Cs3—O25^ii^	81.4 (2)	O10^vi^—O5W—O6W	161.9 (7)
O15—Cs3—O2^ii^	90.2 (2)	O10^vi^—O5W—O7W	104.1 (5)
O15—Cs3—O3W^iii^	141.4 (3)	O10^vi^—O5W—O7W^ix^	88.4 (6)
O15—Cs3—O3W	84.2 (3)	O10^vi^—O5W—O8^ix^	121.4 (6)
O15—Cs3—O10	46.5 (2)	O12^vii^—O5W—W3^vi^	120.8 (5)
O15—Cs3—O13	88.2 (2)	O12^vii^—O5W—Cs5	63.8 (5)
O15—Cs3—O19	46.9 (2)	O12^vii^—O5W—O2^vi^	149.5 (6)
O15—Cs3—O23	51.8 (2)	O12^vii^—O5W—O3W	117.1 (7)
O15—Cs3—O25^ii^	132.2 (2)	O12^vii^—O5W—O4W	74.6 (5)
O19—Cs3—O10	93.3 (2)	O12^vii^—O5W—O6W	97.7 (5)
O23—Cs3—O2^ii^	124.7 (2)	O12^vii^—O5W—O7W	157.4 (6)
O23—Cs3—O3W	89.5 (3)	O12^vii^—O5W—O7W^ix^	130.5 (7)
O23—Cs3—O10	82.5 (2)	O12^vii^—O5W—O8^ix^	78.6 (5)
O23—Cs3—O19	46.2 (2)	O12^vii^—O5W—O10^vi^	97.0 (5)
O23—Cs3—O25^ii^	129.5 (2)	W1—O6—Cs5^xii^	111.3 (6)
O24—Cs3—O2^ii^	49.6 (2)	W1—O6—Cs5^viii^	105.5 (6)
O24—Cs3—O3W	174.4 (3)	W1—O6—Cs5	98.8 (5)
O24—Cs3—O3W^iii^	94.1 (3)	W1—O6—Cs6	98.1 (5)
O24—Cs3—O8	102.3 (3)	Cs5^xii^—O6—Cs5^viii^	97.1 (3)
O24—Cs3—O10	130.1 (2)	Cs5^xii^—O6—Cs5	91.7 (3)
O24—Cs3—O13	51.0 (2)	Cs5^viii^—O6—Cs5	149.0 (4)
O24—Cs3—O15	90.2 (3)	Cs5^xii^—O6—Cs6	149.8 (4)
O24—Cs3—O19	48.6 (2)	Cs5^viii^—O6—Cs6	80.5 (3)
O24—Cs3—O23	87.4 (2)	Cs5—O6—Cs6	77.1 (3)
O24—Cs3—O25^ii^	47.9 (2)	Na1—O6W—Cs8	146.3 (5)
O25^ii^—Cs3—O10	141.4 (2)	Na1—O6W—Na2	89.2 (5)
O25^ii^—Cs3—O19	96.5 (2)	Na1—O6W—O4W	47.4 (4)
O1W^iv^—Cs4—O1W	98.9 (6)	Na1—O6W—O5W	50.9 (4)
O1W^iv^—Cs4—O2W	63.0 (5)	Na1—O6W—O6W^ix^	133.0 (4)
O1W—Cs4—O2W^iv^	59.2 (5)	Na1—O6W—O6W^x^	97.3 (5)
O1W^iv^—Cs4—O2W^iv^	73.8 (5)	Na1—O6W—O7W	43.0 (4)
O1W—Cs4—O2W	72.0 (5)	Na1—O6W—O8^ix^	124.1 (6)
O1W—Cs4—O9	107.3 (5)	Na1—O6W—O8	56.1 (4)
O1W^iv^—Cs4—O9	127.4 (5)	Na1—O6W—O11	95.9 (5)
O1W^iv^—Cs4—O16	116.9 (5)	Na1—O6W—O22^ix^	133.7 (6)
O1W^iv^—Cs4—O17	169.2 (5)	Na2—O6W—Cs8	91.4 (5)
O1W^iv^—Cs4—O18	76.3 (5)	Na2—O6W—O4W	136.3 (6)
O1W^iv^—Cs4—O27^v^	67.7 (5)	Na2—O6W—O5W	89.5 (6)
O2W—Cs4—O2W^iv^	106.5 (4)	Na2—O6W—O6W^ix^	44.9 (3)
O9—Cs4—O2W	82.8 (3)	Na2—O6W—O6W^x^	44.9 (3)
O9—Cs4—O2W^iv^	158.1 (3)	Na2—O6W—O7W	46.3 (4)
O14—Cs4—O1W^iv^	138.9 (5)	Na2—O6W—O8	85.0 (4)
O14—Cs4—O1W	86.2 (5)	Na2—O6W—O8^ix^	83.7 (4)
O14—Cs4—O2W^iv^	74.2 (3)	Na2—O6W—O11	125.6 (6)
O14—Cs4—O2W	152.7 (3)	Na2—O6W—O22^ix^	132.0 (6)
O14—Cs4—O9	88.5 (2)	O4W—O6W—Cs8	120.7 (5)
O14—Cs4—O16	52.9 (3)	O4W—O6W—O6W^x^	123.6 (6)
O14—Cs4—O17	50.3 (2)	O4W—O6W—O6W^ix^	176.3 (6)
O14—Cs4—O18	134.4 (3)	O4W—O6W—O7W	90.0 (5)
O14—Cs4—O27^v^	72.0 (2)	O5W—O6W—Cs8	162.8 (5)
O16—Cs4—O1W	138.3 (5)	O5W—O6W—O4W	68.4 (5)
O16—Cs4—O2W^iv^	109.1 (4)	O5W—O6W—O6W^x^	128.1 (4)
O16—Cs4—O2W	142.5 (4)	O5W—O6W—O6W^ix^	108.8 (5)
O16—Cs4—O9	68.3 (3)	O5W—O6W—O7W	64.4 (4)
O16—Cs4—O17	72.7 (3)	O6W^x^—O6W—Cs8	61.33 (18)
O16—Cs4—O18	88.6 (3)	O6W^ix^—O6W—Cs8	61.33 (18)
O16—Cs4—O27^v^	70.0 (3)	O6W^x^—O6W—O6W^ix^	60.000 (3)
O17—Cs4—O1W	74.4 (5)	O6W^x^—O6W—O7W	65.4 (4)
O17—Cs4—O2W^iv^	108.7 (3)	O6W^ix^—O6W—O7W	90.8 (3)
O17—Cs4—O2W	106.5 (3)	O7W—O6W—Cs8	126.7 (5)
O17—Cs4—O9	49.4 (2)	O8^ix^—O6W—Cs8	89.5 (4)
O17—Cs4—O27^v^	122.2 (3)	O8—O6W—Cs8	90.4 (4)
O18—Cs4—O1W	122.0 (5)	O8^ix^—O6W—O4W	121.8 (5)
O18—Cs4—O2W^iv^	149.7 (3)	O8—O6W—O4W	67.5 (4)
O18—Cs4—O2W	54.1 (3)	O8^ix^—O6W—O5W	73.6 (5)
O18—Cs4—O9	51.1 (2)	O8—O6W—O5W	106.8 (5)
O18—Cs4—O17	99.8 (2)	O8—O6W—O6W^ix^	116.0 (5)
O18—Cs4—O27^v^	121.3 (3)	O8—O6W—O6W^x^	56.1 (5)
O27^v^—Cs4—O1W	108.5 (5)	O8^ix^—O6W—O6W^x^	114.4 (5)
O27^v^—Cs4—O2W	129.9 (3)	O8^ix^—O6W—O6W^ix^	54.5 (5)
O27^v^—Cs4—O2W^iv^	49.4 (3)	O8^ix^—O6W—O7W	111.1 (5)
O27^v^—Cs4—O9	137.6 (2)	O8—O6W—O7W	60.5 (4)
O3W—Cs5—O5	89.7 (2)	O8—O6W—O8^ix^	168.7 (6)
O3W—Cs5—O5W	60.2 (3)	O8—O6W—O11	55.7 (3)
O3W—Cs5—O6	124.3 (3)	O11—O6W—Cs8	57.2 (3)
O3W—Cs5—O12^vii^	103.2 (3)	O11—O6W—O4W	65.3 (4)
O3W—Cs5—O25^vi^	46.6 (3)	O11—O6W—O5W	133.7 (6)
O4—Cs5—O3W	111.1 (3)	O11—O6W—O6W^ix^	117.4 (3)
O4—Cs5—O4W	52.6 (3)	O11—O6W—O6W^x^	80.9 (4)
O4—Cs5—O5	49.5 (2)	O11—O6W—O7W	116.2 (5)
O4—Cs5—O5W	101.7 (3)	O11—O6W—O8^ix^	132.2 (5)
O4—Cs5—O6	78.3 (3)	O22^ix^—O6W—Cs8	65.2 (4)
O4—Cs5—O6^vii^	91.4 (3)	O22^ix^—O6W—O4W	90.5 (5)
O4—Cs5—O10	93.2 (2)	O22^ix^—O6W—O5W	101.9 (5)
O4—Cs5—O12^vii^	76.6 (3)	O22^ix^—O6W—O6W^x^	125.9 (4)
O4—Cs5—O25^vi^	156.2 (3)	O22^ix^—O6W—O6W^ix^	87.7 (5)
O4W—Cs5—O3W	63.4 (3)	O22^ix^—O6W—O7W	164.9 (6)
O4W—Cs5—O5	70.8 (3)	O22^ix^—O6W—O8^ix^	56.4 (4)
O4W—Cs5—O5W	59.7 (3)	O22^ix^—O6W—O8	133.1 (6)
O4W—Cs5—O6	122.7 (3)	O22^ix^—O6W—O11	77.6 (4)
O4W—Cs5—O10	77.2 (3)	W4^ii^—O7—Cs1	92.1 (3)
O4W—Cs5—O12^vii^	68.6 (3)	W4^ii^—O7—Cs2	104.9 (3)
O4W—Cs5—O25^vi^	103.8 (3)	W8—O7—W4^ii^	140.7 (5)
O5—Cs5—O5W	129.4 (3)	W8—O7—Cs1	96.6 (3)
O5—Cs5—O12^vii^	125.2 (3)	W8—O7—Cs2	114.4 (3)
O5—Cs5—O25^vi^	126.0 (2)	Cs2—O7—Cs1	74.17 (18)
O6^vi^—Cs5—O3W	91.3 (3)	Na1—O7W—Na2	85.8 (5)
O6^vii^—Cs5—O3W	140.4 (3)	Na1—O7W—O2^vi^	93.8 (5)
O6^vi^—Cs5—O4	152.5 (3)	Na1—O7W—O2^iii^	172.9 (7)
O6^vi^—Cs5—O4W	153.9 (3)	Na1—O7W—O3W	47.2 (4)
O6^vii^—Cs5—O4W	114.9 (3)	Na1—O7W—O5W	43.6 (4)
O6^vi^—Cs5—O5	118.0 (3)	Na1—O7W—O5W^x^	120.2 (7)
O6^vii^—Cs5—O5	128.6 (3)	Na1—O7W—O6W	42.6 (4)
O6—Cs5—O5	54.1 (2)	Na1—O7W—O6W^x^	88.8 (5)
O6—Cs5—O5W	175.2 (3)	Na1—O7W—O7W^x^	135.2 (5)
O6^vi^—Cs5—O5W	103.4 (3)	Na1—O7W—O7W^ix^	91.3 (7)
O6^vii^—Cs5—O5W	84.2 (3)	Na1—O7W—O8	50.7 (4)
O6^vi^—Cs5—O6	75.8 (3)	Na2—O7W—O2^vi^	114.9 (6)
O6^vii^—Cs5—O6	91.0 (4)	Na2—O7W—O2^iii^	101.2 (5)
O6^vi^—Cs5—O6^vii^	79.9 (4)	Na2—O7W—O3W	132.6 (6)
O6^vi^—Cs5—O10	90.5 (3)	Na2—O7W—O5W	78.3 (5)
O6^vii^—Cs5—O10	167.2 (3)	Na2—O7W—O5W^x^	87.4 (5)
O6^vii^—Cs5—O12^vii^	49.5 (3)	Na2—O7W—O6W^x^	39.6 (4)
O6—Cs5—O12^vii^	131.7 (3)	Na2—O7W—O6W	43.3 (4)
O6^vi^—Cs5—O12^vii^	114.7 (3)	Na2—O7W—O7W^ix^	49.5 (3)
O6^vii^—Cs5—O25^vi^	103.1 (3)	Na2—O7W—O7W^x^	49.5 (3)
O6—Cs5—O25^vi^	119.5 (3)	Na2—O7W—O8	76.3 (5)
O6^vi^—Cs5—O25^vi^	50.5 (3)	O2^vi^—O7W—O2^iii^	83.5 (5)
O10—Cs5—O3W	47.4 (3)	O2^vi^—O7W—O3W	70.0 (4)
O10—Cs5—O5	49.4 (2)	O2^vi^—O7W—O5W^x^	141.3 (6)
O10—Cs5—O5W	106.4 (3)	O2^vi^—O7W—O5W	60.0 (4)
O10—Cs5—O6	78.3 (3)	O2^iii^—O7W—O5W	137.6 (6)
O10—Cs5—O12^vii^	143.4 (3)	O2^iii^—O7W—O6W^x^	96.5 (4)
O10—Cs5—O25^vi^	76.7 (2)	O2^vi^—O7W—O6W^x^	154.2 (6)
O12^vii^—Cs5—O5W	44.3 (3)	O2^vi^—O7W—O6W	111.2 (5)
O25^vi^—Cs5—O5W	62.0 (3)	O2^iii^—O7W—O6W	144.4 (6)
O25^vi^—Cs5—O12^vii^	98.5 (3)	O2^vi^—O7W—O7W^ix^	65.5 (5)
O4^vii^—Cs6—O4	116.98 (11)	O2^vi^—O7W—O7W^x^	102.7 (4)
O4—Cs6—O4^viii^	116.98 (11)	O2^vi^—O7W—O8	143.2 (6)
O4^vii^—Cs6—O4^viii^	116.98 (12)	O3W—O7W—O2^iii^	125.8 (5)
O4^viii^—Cs6—O6^vii^	147.6 (3)	O3W—O7W—O5W	63.8 (4)
O4^viii^—Cs6—O6^viii^	72.1 (3)	O3W—O7W—O6W^x^	127.2 (5)
O4^vii^—Cs6—O6	147.6 (3)	O3W—O7W—O6W	89.8 (4)
O4^vii^—Cs6—O6^vii^	72.1 (3)	O5W^x^—O7W—O2^iii^	60.5 (4)
O4^vii^—Cs6—O6^viii^	79.4 (3)	O5W^x^—O7W—O3W	118.6 (6)
O4—Cs6—O6^viii^	147.6 (3)	O5W^x^—O7W—O5W	158.6 (7)
O4—Cs6—O6	72.1 (3)	O5W^x^—O7W—O6W	106.6 (6)
O4^viii^—Cs6—O6	79.4 (3)	O5W—O7W—O6W^x^	107.3 (5)
O4—Cs6—O6^vii^	79.4 (3)	O5W^x^—O7W—O6W^x^	53.1 (4)
O6^viii^—Cs6—O6^vii^	79.9 (3)	O5W^x^—O7W—O7W^ix^	125.8 (6)
O6—Cs6—O6^vii^	79.9 (3)	O5W^x^—O7W—O7W^x^	67.1 (6)
O6—Cs6—O6^viii^	79.9 (3)	O5W^x^—O7W—O8	70.0 (5)
O26^vii^—Cs6—O4^viii^	161.9 (3)	O6W—O7W—O5W	52.3 (4)
O26^viii^—Cs6—O4	161.9 (3)	O6W—O7W—O6W^x^	56.1 (5)
O26—Cs6—O4^viii^	68.6 (2)	O7W^x^—O7W—O2^iii^	51.8 (4)
O26—Cs6—O4	50.5 (2)	O7W^ix^—O7W—O2^iii^	93.4 (4)
O26—Cs6—O4^vii^	161.9 (3)	O7W^ix^—O7W—O3W	115.1 (7)
O26^vii^—Cs6—O4	68.6 (2)	O7W^x^—O7W—O3W	172.7 (5)
O26^viii^—Cs6—O4^vii^	68.6 (2)	O7W^x^—O7W—O5W	112.8 (6)
O26^vii^—Cs6—O4^vii^	50.5 (2)	O7W^ix^—O7W—O5W	53.8 (6)
O26^viii^—Cs6—O4^viii^	50.5 (2)	O7W^x^—O7W—O6W	92.8 (3)
O26^vii^—Cs6—O6	118.2 (3)	O7W^x^—O7W—O6W^x^	59.5 (3)
O26^vii^—Cs6—O6^vii^	47.7 (2)	O7W^ix^—O7W—O6W	66.5 (4)
O26^viii^—Cs6—O6	113.0 (3)	O7W^ix^—O7W—O6W^x^	88.7 (3)
O26^viii^—Cs6—O6^viii^	47.7 (2)	O7W^ix^—O7W—O7W^x^	60.000 (6)
O26^vii^—Cs6—O6^viii^	113.0 (3)	O8—O7W—O2^iii^	130.6 (5)
O26—Cs6—O6^viii^	118.2 (3)	O8—O7W—O3W	77.0 (4)
O26—Cs6—O6	47.7 (2)	O8—O7W—O5W	91.0 (5)
O26—Cs6—O6^vii^	113.0 (3)	O8—O7W—O6W	51.6 (3)
O26^viii^—Cs6—O6^vii^	118.2 (3)	O8—O7W—O6W^x^	50.4 (3)
O26^viii^—Cs6—O26	119.12 (6)	O8—O7W—O7W^x^	109.8 (4)
O26—Cs6—O26^vii^	119.12 (6)	O8—O7W—O7W^ix^	117.2 (4)
O26^viii^—Cs6—O26^vii^	119.11 (6)	W7—O8—Cs3	103.1 (4)
O2W^vii^—Cs7—O13^ix^	70.8 (3)	W7—O8—Na1	139.4 (6)
O4—Cs7—O2W^vii^	135.2 (3)	W7—O8—Na2	127.0 (5)
O4—Cs7—O4W	52.0 (3)	W7—O8—O4W	97.0 (5)
O4—Cs7—O12^vii^	83.3 (3)	W7—O8—O5W^x^	121.6 (5)
O4—Cs7—O13^ix^	144.0 (2)	W7—O8—O6W^x^	95.9 (5)
O4—Cs7—O16	85.4 (3)	W7—O8—O6W	107.4 (5)
O4—Cs7—O21^ix^	126.3 (3)	W7—O8—O7W	168.3 (6)
O4W—Cs7—O2W^vii^	165.7 (4)	Cs3—O8—Na2	129.1 (4)
O4W—Cs7—O13^ix^	110.0 (3)	Cs3—O8—O4W	86.7 (4)
O12^vii^—Cs7—O2W^vii^	119.3 (3)	Cs3—O8—O5W^x^	83.2 (3)
O12^vii^—Cs7—O4W	71.0 (3)	Cs3—O8—O7W	86.8 (4)
O12^vii^—Cs7—O13^ix^	60.6 (3)	Na1—O8—Cs3	86.4 (3)
O12^vii^—Cs7—O16	140.7 (3)	Na1—O8—Na2	62.6 (3)
O12^vii^—Cs7—O21^ix^	70.0 (3)	Na1—O8—O4W	43.6 (3)
O16—Cs7—O2W^vii^	94.8 (3)	Na1—O8—O5W^x^	98.5 (4)
O16—Cs7—O4W	72.4 (3)	Na1—O8—O6W	50.4 (3)
O16—Cs7—O13^ix^	121.7 (3)	Na1—O8—O6W^x^	103.5 (4)
O16—Cs7—O21^ix^	142.7 (3)	Na1—O8—O7W	46.3 (3)
O21^ix^—Cs7—O2W^vii^	49.7 (3)	Na2—O8—O5W^x^	64.6 (3)
O21^ix^—Cs7—O4W	140.8 (3)	O4W—O8—Na2	94.8 (3)
O21^ix^—Cs7—O13^ix^	44.2 (2)	O4W—O8—O5W^x^	141.3 (5)
O22^ix^—Cs7—O2W^vii^	89.0 (3)	O6W^x^—O8—Cs3	137.1 (4)
O22^ix^—Cs7—O4	134.3 (3)	O6W—O8—Cs3	136.4 (4)
O22^ix^—Cs7—O4W	82.3 (3)	O6W^x^—O8—Na2	40.9 (3)
O22^ix^—Cs7—O12^vii^	83.1 (3)	O6W—O8—Na2	41.0 (3)
O22^ix^—Cs7—O13^ix^	46.5 (2)	O6W—O8—O4W	59.6 (4)
O22^ix^—Cs7—O16	78.3 (3)	O6W^x^—O8—O4W	129.0 (5)
O22^ix^—Cs7—O21^ix^	88.8 (3)	O6W^x^—O8—O5W^x^	54.3 (4)
O26^vii^—Cs7—O2W^vii^	88.7 (3)	O6W—O8—O5W^x^	105.5 (4)
O26^vii^—Cs7—O4	76.1 (3)	O6W—O8—O6W^x^	69.4 (6)
O26^vii^—Cs7—O4W	105.6 (3)	O6W^x^—O8—O7W	72.4 (4)
O26^vii^—Cs7—O12^vii^	51.6 (3)	O6W—O8—O7W	67.9 (4)
O26^vii^—Cs7—O13^ix^	81.3 (2)	O7W—O8—Na2	42.4 (4)
O26^vii^—Cs7—O16	156.6 (3)	O7W—O8—O4W	89.9 (4)
O26^vii^—Cs7—O21^ix^	50.6 (2)	O7W—O8—O5W^x^	52.5 (4)
O26^vii^—Cs7—O22^ix^	124.9 (3)	W2—O9—W4	156.8 (5)
O6W^ix^—Cs8—O6W^x^	57.3 (4)	W2—O9—Cs4	96.3 (3)
O6W^ix^—Cs8—O6W	57.3 (4)	W2—O9—O4W	92.5 (4)
O6W^x^—Cs8—O6W	57.3 (4)	W4—O9—Cs4	100.5 (3)
O11^x^—Cs8—O6W^ix^	76.5 (3)	W4—O9—O4W	92.8 (4)
O11—Cs8—O6W^x^	76.5 (3)	O4W—O9—Cs4	120.1 (4)
O11^x^—Cs8—O6W	109.3 (3)	W3—O10—Cs3	91.7 (4)
O11—Cs8—O6W^ix^	109.3 (3)	W3—O10—Cs5	116.1 (4)
O11^ix^—Cs8—O6W	76.5 (3)	W3—O10—O3W	146.2 (6)
O11^ix^—Cs8—O6W^x^	109.3 (3)	W3—O10—O5W^xii^	106.7 (5)
O11—Cs8—O6W	52.9 (3)	Cs5—O10—Cs3	115.3 (3)
O11^x^—Cs8—O6W^x^	52.9 (3)	O3W—O10—Cs3	59.8 (3)
O11^ix^—Cs8—O6W^ix^	52.9 (3)	O3W—O10—Cs5	67.9 (3)
O11^ix^—Cs8—O11^x^	116.01 (11)	O3W—O10—O5W^xii^	98.9 (5)
O11^x^—Cs8—O11	116.00 (11)	O5W^xii^—O10—Cs3	106.0 (5)
O11^ix^—Cs8—O11	116.00 (11)	O5W^xii^—O10—Cs5	117.7 (5)
O11^x^—Cs8—O20^ix^	116.3 (3)	W5—O11—W7	109.6 (4)
O11^ix^—Cs8—O20^ix^	52.9 (2)	W5—O11—Cs8	102.5 (3)
O11—Cs8—O20^ix^	124.0 (3)	W5—O11—O4W	113.9 (5)
O11^ix^—Cs8—O20	116.3 (3)	W5—O11—O6W	151.2 (5)
O11^ix^—Cs8—O20^x^	124.0 (3)	W7—O11—Cs8	107.4 (4)
O11—Cs8—O20	52.9 (2)	W7—O11—O4W	94.5 (4)
O11^x^—Cs8—O20^x^	52.9 (2)	W7—O11—O6W	99.1 (4)
O11—Cs8—O20^x^	116.3 (3)	Cs8—O11—O4W	127.8 (4)
O11^x^—Cs8—O20	124.0 (3)	O6W—O11—Cs8	69.8 (3)
O11^ix^—Cs8—O22	151.3 (3)	O6W—O11—O4W	60.1 (4)
O11—Cs8—O22^x^	151.3 (3)	W1—O12—Cs5^viii^	94.0 (6)
O11^x^—Cs8—O22^ix^	151.3 (2)	W1—O12—Cs7^viii^	104.5 (5)
O11^x^—Cs8—O22	66.4 (2)	W1—O12—O4W^viii^	135.5 (7)
O11^ix^—Cs8—O22^ix^	50.1 (2)	W1—O12—O5W^viii^	142.2 (8)
O11—Cs8—O22	50.1 (2)	Cs5^viii^—O12—O4W^viii^	52.6 (3)
O11—Cs8—O22^ix^	66.4 (3)	Cs7^viii^—O12—Cs5^viii^	93.5 (4)
O11^ix^—Cs8—O22^x^	66.4 (2)	Cs7^viii^—O12—O4W^viii^	57.1 (3)
O11^x^—Cs8—O22^x^	50.1 (2)	O5W^viii^—O12—Cs5^viii^	71.9 (5)
O20^ix^—Cs8—O6W^ix^	101.0 (3)	O5W^viii^—O12—Cs7^viii^	111.1 (5)
O20^x^—Cs8—O6W^x^	101.0 (3)	O5W^viii^—O12—O4W^viii^	61.9 (5)
O20^x^—Cs8—O6W	156.1 (3)	W7—O13—W8	156.6 (5)
O20^x^—Cs8—O6W^ix^	122.3 (3)	W7—O13—Cs3	94.1 (3)
O20^ix^—Cs8—O6W	122.3 (3)	W7—O13—Cs7^x^	90.6 (3)
O20—Cs8—O6W^ix^	156.1 (3)	W8—O13—Cs3	95.1 (3)
O20—Cs8—O6W	101.0 (3)	W8—O13—Cs7^x^	101.6 (3)
O20^ix^—Cs8—O6W^x^	156.1 (3)	Cs3—O13—Cs7^x^	125.0 (3)
O20—Cs8—O6W^x^	122.3 (3)	W5—O14—W6	110.7 (4)
O20^x^—Cs8—O20	81.6 (3)	W5—O14—Cs4	102.4 (4)
O20^ix^—Cs8—O20^x^	81.6 (3)	W5—O14—Cs9^v^	102.8 (4)
O20^ix^—Cs8—O20	81.6 (3)	W6—O14—Cs4	112.4 (4)
O20^ix^—Cs8—O22^x^	81.5 (3)	W6—O14—Cs9^v^	121.4 (4)
O20^x^—Cs8—O22^x^	77.3 (2)	Cs9^v^—O14—Cs4	105.2 (3)
O20—Cs8—O22^x^	154.6 (3)	W3—O15—W4	152.3 (5)
O20—Cs8—O22^ix^	81.5 (3)	W3—O15—Cs1	95.4 (3)
O20^x^—Cs8—O22	81.5 (3)	W3—O15—Cs3	109.4 (3)
O20^ix^—Cs8—O22^ix^	77.3 (3)	W4—O15—Cs1	95.8 (3)
O20—Cs8—O22	77.3 (2)	W4—O15—Cs3	96.3 (3)
O20^x^—Cs8—O22^ix^	154.6 (3)	Cs3—O15—Cs1	87.9 (2)
O20^ix^—Cs8—O22	154.6 (3)	W5—O16—Cs4	103.7 (5)
O22^x^—Cs8—O6W^ix^	47.2 (3)	W5—O16—Cs7	137.7 (5)
O22—Cs8—O6W	76.0 (3)	W5—O16—Cs9^ix^	97.3 (5)
O22^ix^—Cs8—O6W^x^	104.1 (3)	W5—O16—O4W	95.4 (5)
O22^ix^—Cs8—O6W^ix^	76.0 (3)	Cs4—O16—Cs7	114.3 (3)
O22^x^—Cs8—O6W^x^	76.0 (3)	Cs4—O16—Cs9^ix^	124.9 (4)
O22^x^—Cs8—O6W	104.1 (3)	Cs4—O16—O4W	110.2 (4)
O22—Cs8—O6W^ix^	104.1 (3)	Cs7—O16—O4W	55.4 (3)
O22^ix^—Cs8—O6W	47.2 (3)	Cs9^ix^—O16—Cs7	76.0 (2)
O22—Cs8—O6W^x^	47.1 (3)	Cs9^ix^—O16—O4W	117.8 (4)
O22^ix^—Cs8—O22	112.90 (16)	W4—O17—W6	137.1 (5)
O22^x^—Cs8—O22^ix^	112.91 (16)	W4—O17—Cs2	91.0 (3)
O22^x^—Cs8—O22	112.91 (16)	W4—O17—Cs4	116.9 (4)
O1—Cs9—O20	53.3 (2)	W6—O17—Cs2	86.7 (3)
O2W^ii^—Cs9—O1	75.3 (3)	W6—O17—Cs4	103.2 (3)
O2W^ii^—Cs9—O20	125.6 (3)	Cs4—O17—Cs2	113.0 (3)
O14^xi^—Cs9—Cs7^x^	117.02 (18)	W2—O18—Cs4	108.7 (4)
O14^xi^—Cs9—O1	127.3 (2)	W2—O18—Na3	150.2 (8)
O14^xi^—Cs9—O2W^ii^	76.3 (3)	W2—O18—O1W^iv^	159.9 (6)
O14^xi^—Cs9—O16^x^	103.8 (3)	W2—O18—O2W	117.7 (5)
O14^xi^—Cs9—O20	119.4 (2)	Cs4—O18—O1W^iv^	51.2 (4)
O14^xi^—Cs9—O20^x^	108.4 (3)	Na3—O18—Cs4	91.4 (6)
O14^xi^—Cs9—O20^xi^	53.6 (2)	Na3—O18—O1W^iv^	45.3 (7)
O14^xi^—Cs9—O22	161.9 (3)	Na3—O18—O2W	89.8 (7)
O16^x^—Cs9—O1	123.4 (2)	O2W—O18—Cs4	68.3 (4)
O16^x^—Cs9—O2W^ii^	98.0 (3)	O2W—O18—O1W^iv^	58.6 (5)
O16^x^—Cs9—O20^xi^	117.0 (3)	W4—O19—W8	141.3 (5)
O16^x^—Cs9—O20	123.1 (2)	W4—O19—Cs1	113.8 (4)
O16^x^—Cs9—O22	74.7 (3)	W4—O19—Cs2	99.5 (3)
O20^x^—Cs9—O1	117.9 (2)	W4—O19—Cs3	91.1 (3)
O20^xi^—Cs9—O1	112.7 (2)	W8—O19—Cs1	104.6 (3)
O20^x^—Cs9—O2W^ii^	151.0 (3)	W8—O19—Cs2	95.1 (3)
O20^xi^—Cs9—O2W^ii^	123.1 (4)	W8—O19—Cs3	85.6 (3)
O20^x^—Cs9—O16^x^	53.0 (3)	Cs1—O19—Cs2	75.56 (19)
O20^x^—Cs9—O20^xi^	77.6 (3)	Cs1—O19—Cs3	86.5 (2)
O20^xi^—Cs9—O20	70.9 (3)	Cs2—O19—Cs3	161.6 (3)
O20^x^—Cs9—O20	78.1 (4)	W5—O20—Cs8	104.0 (4)
O20^x^—Cs9—O22	85.4 (3)	W5—O20—Cs9	96.9 (4)
O20^xi^—Cs9—O22	143.5 (3)	W5—O20—Cs9^v^	99.4 (4)
O22—Cs9—O1	49.0 (2)	W5—O20—Cs9^ix^	102.1 (5)
O22—Cs9—O2W^ii^	86.0 (3)	Cs8—O20—Cs9	81.4 (3)
O22—Cs9—O20	74.1 (3)	Cs8—O20—Cs9^v^	154.6 (3)
O27—Cs9—O1	52.0 (2)	Cs9^ix^—O20—Cs8	89.6 (3)
O27—Cs9—O2W^ii^	52.3 (3)	Cs9^ix^—O20—Cs9^v^	95.1 (3)
O27—Cs9—O14^xi^	75.4 (3)	Cs9^ix^—O20—Cs9	160.4 (3)
O27—Cs9—O16^x^	150.0 (3)	Cs9^v^—O20—Cs9	86.2 (2)
O27—Cs9—O20	80.0 (3)	W2^ii^—O21—Cs7^x^	94.8 (3)
O27—Cs9—O20^x^	156.5 (3)	W2^ii^—O21—O2W^ii^	104.9 (5)
O27—Cs9—O20^xi^	87.2 (3)	W8—O21—W2^ii^	135.2 (5)
O27—Cs9—O22	96.7 (3)	W8—O21—Cs7^x^	122.6 (4)
O3W—Na1—O5W	95.8 (5)	W8—O21—O2W^ii^	111.3 (5)
O3W—Na1—O8	110.6 (4)	O2W^ii^—O21—Cs7^x^	67.8 (4)
O4W—Na1—O3W	94.9 (5)	W7—O22—Cs7^x^	122.0 (5)
O4W—Na1—O5W	95.0 (6)	W7—O22—Cs8	103.1 (5)
O4W—Na1—O6W	85.0 (5)	W7—O22—Cs9	115.3 (5)
O4W—Na1—O7W	171.0 (6)	W7—O22—O6W^x^	103.8 (5)
O4W—Na1—O8	88.2 (5)	Cs7^x^—O22—Cs8	134.8 (3)
O5W—Na1—O8	153.1 (5)	Cs7^x^—O22—Cs9	77.9 (3)
O6W—Na1—O3W	175.9 (5)	Cs9—O22—Cs8	81.7 (2)
O6W—Na1—O5W	80.1 (5)	O6W^x^—O22—Cs7^x^	100.5 (4)
O6W—Na1—O7W	94.4 (6)	O6W^x^—O22—Cs8	67.7 (4)
O6W—Na1—O8	73.5 (4)	O6W^x^—O22—Cs9	134.8 (4)
O7W—Na1—O3W	86.4 (5)	W4—O23—W7	136.2 (5)
O7W—Na1—O5W	93.7 (6)	W4—O23—Cs3	100.0 (4)
O7W—Na1—O8	83.0 (5)	W4—O23—O4W	118.0 (5)
O6W^ix^—Na2—O6W	90.3 (6)	W7—O23—Cs3	85.9 (3)
O6W^ix^—Na2—O6W^x^	90.3 (6)	W7—O23—O4W	104.6 (4)
O6W^x^—Na2—O6W	90.3 (6)	O4W—O23—Cs3	94.0 (4)
O6W^x^—Na2—O7W^ix^	171.5 (6)	W3^ii^—O24—Cs1	83.5 (3)
O6W^ix^—Na2—O7W	171.5 (6)	W3^ii^—O24—Cs3	106.8 (3)
O6W—Na2—O7W	90.4 (4)	W8—O24—W3^ii^	141.0 (5)
O6W—Na2—O7W^ix^	98.2 (5)	W8—O24—Cs1	95.1 (4)
O6W^x^—Na2—O7W	98.2 (5)	W8—O24—Cs3	112.1 (4)
O6W^ix^—Na2—O7W^x^	98.2 (5)	Cs3—O24—Cs1	87.6 (2)
O6W^ix^—Na2—O7W^ix^	90.4 (4)	W1—O25—W3	109.4 (4)
O6W—Na2—O7W^x^	171.5 (6)	W1—O25—Cs3^i^	113.9 (4)
O6W^x^—Na2—O7W^x^	90.4 (4)	W1—O25—Cs5^xii^	93.4 (3)
O7W^ix^—Na2—O7W	81.1 (6)	W1—O25—O3W^xii^	131.2 (5)
O7W^ix^—Na2—O7W^x^	81.1 (6)	W1—O25—O5W^xii^	142.8 (5)
O7W^x^—Na2—O7W	81.1 (6)	W3—O25—Cs3^i^	91.5 (3)
O1W^iv^—Na3—W2	109.9 (8)	W3—O25—Cs5^xii^	127.3 (4)
O1W^iv^—Na3—O2W	60.7 (7)	W3—O25—O3W^xii^	118.8 (5)
O18—Na3—O1W^iv^	98.0 (10)	W3—O25—O5W^xii^	72.6 (4)
O18—Na3—O2W	52.6 (6)	Cs3^i^—O25—O5W^xii^	103.0 (3)
W5—O1—W6	104.1 (4)	Cs5^xii^—O25—Cs3^i^	122.3 (3)
W5—O1—W7	103.1 (4)	Cs5^xii^—O25—O5W^xii^	61.8 (3)
W5—O1—Cs9	95.8 (3)	O3W^xii^—O25—Cs3^i^	59.7 (3)
W6—O1—Cs9	93.0 (3)	O3W^xii^—O25—Cs5^xii^	64.0 (3)
W7—O1—W6	147.5 (4)	O3W^xii^—O25—O5W^xii^	65.0 (4)
W7—O1—Cs9	101.4 (3)	W1—O26—W2	111.1 (4)
W6^i^—O1W—Cs2	67.1 (4)	W1—O26—Cs6	113.5 (4)
W6^i^—O1W—O2W	58.2 (4)	W1—O26—Cs7^viii^	107.4 (4)
W6^i^—O1W—O18^iv^	117.1 (6)	W2—O26—Cs6	102.8 (4)
Cs2—O1W—O2W	102.1 (6)	W2—O26—Cs7^viii^	115.4 (4)
Cs4—O1W—W6^i^	108.4 (7)	Cs7^viii^—O26—Cs6	106.7 (3)
Cs4^iv^—O1W—W6^i^	85.4 (6)	W6—O27—Cs4^xi^	138.2 (5)
Cs4^iv^—O1W—Cs2	152.4 (8)	W6—O27—Cs9	118.5 (5)
Cs4—O1W—Cs2	104.7 (6)	W6—O27—O1W^ii^	84.5 (6)
Cs4^iv^—O1W—Cs4	81.1 (6)	W6—O27—O2W^ii^	108.6 (5)
Cs4—O1W—O2W^iv^	63.2 (5)	Cs4^xi^—O27—O1W^ii^	54.1 (4)
Cs4—O1W—O2W	55.0 (4)	Cs9—O27—Cs4^xi^	101.5 (3)
Cs4^iv^—O1W—O2W	58.6 (5)	Cs9—O27—O1W^ii^	145.7 (5)
Cs4^iv^—O1W—O2W^iv^	63.8 (6)	O2W^ii^—O27—Cs4^xi^	71.3 (4)
Cs4^iv^—O1W—O3^i^	97.3 (7)	O2W^ii^—O27—Cs9	71.6 (5)
Cs4—O1W—O3^i^	84.7 (6)	O2W^ii^—O27—O1W^ii^	77.2 (6)
Cs4^iv^—O1W—O18^iv^	52.5 (4)	W6—O28—Cs2	107.5 (4)
Cs4—O1W—O18^iv^	108.0 (7)	W6—O28—O1W^ii^	102.8 (6)
Cs4—O1W—O27^i^	99.0 (7)	W6—O28—O1W	144.1 (7)
Cs4^iv^—O1W—O27^i^	58.2 (5)	O1W^ii^—O28—Cs2	76.2 (5)
Na3^iv^—O1W—W6^i^	110.0 (9)	O1W—O28—Cs2	79.6 (6)
Na3^iv^—O1W—Cs2	106.1 (8)	O1W—O28—O1W^ii^	113.0 (10)

Symmetry codes: (i) −*y*+1, *x*−*y*+1, *z*; (ii) −*x*+*y*, −*x*+1, *z*; (iii) −*x*+1, −*y*+1, −*z*+1; (iv) −*x*+4/3, −*y*+5/3, −*z*+2/3; (v) *y*+1/3, −*x*+*y*+2/3, −*z*+2/3; (vi) *x*−*y*+1, *x*, −*z*+1; (vii) −*y*+2, *x*−*y*+1, *z*; (viii) −*x*+*y*+1, −*x*+2, *z*; (ix) −*x*+*y*+1, −*x*+1, *z*; (x) −*y*+1, *x*−*y*, *z*; (xi) *x*−*y*+1/3, *x*−1/3, −*z*+2/3; (xii) *y*, −*x*+*y*+1, −*z*+1.

## References

[bb1] Antonio, M., Nyman, M. & Anderson, T. (2009). *Angew. Chem. Int. Ed.* **48**, 6136–6140. 10.1002/anie.200805323 19396852

[bb2] Brüdgam, I., Fuchs, J., Hartl, H. & Palm, R. (1998). *Angew. Chem. Int. Ed.* **37**, 2668–2671. 10.1002/(SICI)1521-3773(19981016)37:19<2668::AID-ANIE2668>3.0.CO;2-8 29711599

[bb3] Dolomanov, O. V., Bourhis, L. J., Gildea, R. J., Howard, J. A. K. & Puschmann, H. (2009). *J. Appl. Cryst.* **42**, 339–341.

[bb4] Groom, C. R., Bruno, I. J., Lightfoot, M. P. & Ward, S. C. (2016). *Acta Cryst.* B**72**, 171–179. 10.1107/S2052520616003954 PMC4822653 27048719

[bb5] Iftikhar, T., Izarova, N. V. & Kögerler, P. (2024). *Inorg. Chem.* **63**, 99–107. 10.1021/acs.inorgchem.3c01051 38111082

[bb6] Liu, Y.-J., Jin, M.-T., Chen, L.-J. & Zhao, J.-W. (2018). *Acta Cryst.* C**74**, 1202–1221. 10.1107/S2053229618012524 30398172

[bb7] Massa, W. & Gould, R. O. (2004). *Crystal Structure Determination*, 2nd ed., p. 123. New York: Springer-Verlag.

[bb8] Misra, A., Kozma, K., Streb, C. & Nyman, M. (2020). *Angew. Chem. Int. Ed.* **59**, 596–612. 10.1002/anie.201905600 PMC6972580 31260159

[bb9] Pope, M. T. (1983). *Heteropoly and Isopoly Oxometalates*,1st ed., p. 33. Berlin, Heidelberg: Springer.

[bb10] Rigaku OD (2019). *CrysAlis PRO*. Rigaku Oxford Diffraction, Yarnton, England.

[bb11] Sheldrick, G. M. (2015*a*). *Acta Cryst.* A**71**, 3–8.

[bb12] Sheldrick, G. M. (2015*b*). *Acta Cryst.* C**71**, 3–8.

[bb13] Wang, M., Pang, J., Wang, J. & Niu, J. (2024). *Coord. Chem. Rev.* **508**, 215730–215757.

[bb14] Zagorac, D., Müller, H., Ruehl, S., Zagorac, J. & Rehme, S. (2019). *J. Appl. Cryst.* **52**, 918–925. 10.1107/S160057671900997X PMC6782081 31636516

